# Exploring skeletal disorders in cattle and sheep: a WGS-based framework for diagnosis and classification

**DOI:** 10.1186/s12711-025-01002-z

**Published:** 2025-09-25

**Authors:** Joana Jacinto, Anna Letko, Arcangelo Gentile, Arthur Otter, Tobias Floyd, Rachael Collins, Moyna Richey, Helen Carty, Sandra Scholes, Alwyn Jones, Harriet Fuller, Irene M. Häfliger, Ben Strugnell, Eveline Studer, Cinzia Benazzi, Marilena Bolcato, Jože Starič, Alessia Diana, Jim Weber, Markus Freick, Gesine Lühken, Imke Tammen, David C. E. Kraft, Celina M. Lindgren, Marlene Sickinger, Sara Soto, Brendon A. O’Rourke, Jørgen S. Agerholm, Cord Drögemüller

**Affiliations:** 1 Clinic for Ruminants, Vetsuisse Faculty, University of Bern, CH-3012 Bern, Switzerland; 2 Institute of Genetics, Vetsuisse Faculty, University of Bern, CH-3012 Bern, Switzerland; 3 Department of Veterinary Medical Sciences, University of Bologna, Ozzano Dell’Emilia (Bologna), 40064 Bologna, Italy; 4 Animal and Plant Health Agency, Addlestone, UK; 5 SRUC Veterinary and Analytical Services, Pentlands Science Park, Bush Estate Loan, Midlothian, Penicuik, EH26 0PZ UK; 6 Sheep & Beef Health Services, Ivington, Leominster, HR6 0JH UK; 7 Farm Post Mortems Ltd, Hamsterley, Bishop Auckland, County Durham, DL13 3QF UK; 8 Clinic for Reproduction and Large Animals, Veterinary Faculty, University of Ljubljana, 1000 Ljubljana, Slovenia; 9 Institute of Agricultural and Nutritional Sciences, Martin Luther University Halle-Wittenberg, 06120 Halle (Saale), Germany; 10 Institute of Animal Breeding and Genetics, Justus Liebig University Giessen, 35390 Giessen, Germany; 11 Sydney School of Veterinary Science, Faculty of Science, The University of Sydney, Sydney, NSW 2006 Australia; 12 Department of Dentistry and Oral Health, Aarhus University, Aarhus, Denmark; 13 Department of Veterinary Clinical Sciences, Faculty of Health and Medical Sciences, University of Copenhagen, 2630 Taastrup, Denmark; 14 Clinic for Ruminants and Herd Health Management, Justus Liebig University Giessen, 35392 Giessen, Germany; 15 Institute of Animal Pathology, Vetsuisse Faculty, University of Bern, Länggassstrasse 122, 3012 Bern, Switzerland; 16 Department of Primary Industries and Regional Development, Elizabeth Macarthur Agricultural Institute, Woodbridge Road, Menangle, NSW 2568 Australia; 17 PMB4008, Narellan, NSW 2567 Australia

## Abstract

**Background:**

Genetic skeletal disorders are a heterogeneous group of syndromic or non-syndromic diseases characterized by abnormal bone, joint or cartilage development. These disorders generally occur sporadically in ruminants. Although a genetic etiology is often suspected, only a limited number of causal variants have been identified and no comprehensive genetic analyses of a cohort of bovine and ovine skeletal developmental defects have been published. The aims of our study were (1) to propose a nosology of genetic skeletal disorders in cattle and sheep and (2) to contribute to the nosology with a number of novel genomically characterized cases.

**Results:**

Based on a literature review, the proposed nosology of skeletal disorders in cattle and sheep with a confirmed molecular cause was found to comprise 43 different disorders associated with 45 different genes. In addition, horn traits were also included. The disorders were grouped into 21 categories based on the human medical nosology. Thirty novel bovine and nine ovine cases of congenital skeletal disorders were investigated. These represented 19 different disorders, which were grouped into 9 categories. Whole-genome sequencing (WGS) data were generated based on sample availability for either complete trios, affected paternal halfsiblings or isolated single cases. We identified 21 SNVs or small indels for 12 skeletal disorders. Of these, 17 were considered candidate variants affecting 16 different genes, including 11 that were classified as pathogenic and six as likely pathogenic. Additionally, the remaining 4 SNVs were of uncertain significance. Two aneuploidies (trisomy and partial monosomy) were the cause of two different disorders. For eight cases affected by six disorders no variant could be identified. Different modes of inheritance were detected, including spontaneous dominant de novo mutations, autosomal recessive alleles, an X-linked dominant allele, as well as aneuploidies. The overall molecular genetic diagnostic rate was 64%.

**Conclusions:**

Genomic analysis revealed considerable heterogeneity of the described phenotypes in terms of mode of inheritance, affected genes, and variant type. We propose, for the first time in veterinary medicine, a nosology of genetic skeletal disorders in ruminants that may be useful for more precise differential clinicopathological diagnosis. We emphasize the potential of WGS to enhance genetic disease diagnosis and the importance of adopting a nosology for disease categorization.

**Supplementary Information:**

The online version contains supplementary material available at 10.1186/s12711-025-01002-z.

## Background

Genetic disorders are considered individually rare; however, in humans they account for approximately 80% of rare disorders, of which there are many thousands [1]. Most rare disorders show a Mendelian inheritance, particularly those being expressed at an early developmental stage. This is mostly due to changes in the function of a single gene, resulting from pathogenic variants. There is no generally accepted definition of a rare disorder in veterinary medicine. Nevertheless, in human medicine, the mean prevalence threshold is below 50 cases per 100,000 individuals [2]. Similarly, the same definition may be applied to domestic animals such as cattle and sheep. However, it should be noted that the diagnosis of rare disorders in farm animals has historically been poorly documented, and therefore no precise data is available.

Some of the first reports of Mendelian lethal disorders in livestock were published in 1928, several decades before the structure of DNA was reported [3, 4]. Beyond clinical description and pathological examination, the underlying molecular causes have long remained largely unknown, despite a suggested hereditary etiology based on pedigree data. In the last two decades, molecular genetics has evolved tremendously, especially with the development of second- and third-generation sequencing technologies that allow rapid and cost-effective whole-genome sequencing (WGS) [5]. The decoding of the bovine genome in 2009 and the ovine genome in 2014 allowed the creation of the first draft of the bovine genome assembly from an inbred Hereford cow and the ovine assembly from two Texel sheep [6, 7]. In addition to the methodological enhancements, the accessibility of these reference sequences marked a significant milestone for studying genetic disorders in domestic ruminants [5, 8]. Due to the persistent advancement of molecular genetic methods, the detailed case characterization and the active participation of stakeholders, more types of genotype–phenotype associations are now investigated.

It is widely acknowledged that the extensive use of a limited number of sires in livestock breeding can result in an increase in the number of animals born with recessive genetic defects. This is largely due to the concomitant increase in co-ancestry and inbreeding, while facilitating straightforward molecular analysis [9]. Moreover, it has been demonstrated that the capacity to conduct WGS facilitates the identification of the underlying causes of sporadic dominant Mendelian disorders in cattle [10]. The Online Mendelian Inheritance in Animals (OMIA) database, which catalogues inherited disorders, other single-locus traits, and associated genes and variants in animals, reports that there are more than 270 known Mendelian diseases in taurine cattle and more than 90 in sheep, with at least one known likely causal variant for 70 and 50 percent, respectively (accessed 03.01.2025) [11].

Disease nosologies have been established for various groups of human disorders. The adoption of this categorization of diseases is essential for several reasons, including standardization by providing a common terminology for healthcare professionals and, improved diagnosis, treatment, and prognosis by using an accurate classification through faster disease identification, appropriate treatment, and prognosis establishment [12–14]. Research and epidemiology based on a defined nosological framework also help scientists to systematically categorize diseases, improve understanding of their causes, and estimate their frequency in populations; and education and training by creating a fundamental aspect of medical education, helping professionals to understand diseases systematically [12–14]. In veterinary medicine, similar initiatives and classification frameworks are emerging to parallel human nosologies [15, 16]. Integrating veterinary and human nosologies enhance diagnosis, interdisciplinary collaboration and translational research.

Genetic skeletal disorders are a broad group of rare disorders involving abnormal bone, joint or cartilage development leading to abnormal size and structure of the skeleton [17, 18]. In human medicine, the “Nosology of genetic skeletal disorders” including more than 771 different skeletal disorders that range in severity from mild to lethal forms. These disorders are categorized into 41 distinct groups and have been linked with pathogenic variants affecting over 550 genes with diverse functional roles and different modes of inheritance [19]. Several genetic skeletal disorders have been observed in animals demonstrating the need for a comparable classification system that organizes these disorders systematically. At present, there is no proposed nosology for genetic skeletal disorders in veterinary medicine. In cattle, the majority of genetic skeletal disorders characterized to the molecular level are lethal and syndromic (Table 1). Nevertheless, some non-lethal disorders have also been documented. In sheep, the number of described genetic skeletal disorders and associated pathogenic variants is considerably lower than in cattle, with most being recessive (Table 1).

**Table 1:** The nosology of genetic skeletal disorders in cattle and sheep with a known molecular cause: categorization, mode of inheritance, associated genes and pathogenic variants

Category*	Skeletal disorder	Species	Breed	Gene	MOI	Variant(s) type	OMIA ID	Reference(s)
*FGFR3*-related chondrodysplasias	Chondrodysplasia	*Bos taurus*	Holstein	*FGFR3*	AD	Nonsense	001703–9913	[140]
	Chondrodysplasia (SLS)	*Ovis aries*	Suffolk	*FGFR3*	AR	Missense	001703–9940	[141]
Type 2 collagen disorders	Achondrogenesis, type II	*Bos taurus*	Holstein, crossbred	*COL2A1*	AD	Splice site, missense, large deletion	001926–9913	[10, 48, 52–54]
Osteogenesis imperfecta and bone fragility group	Osteogenesis imperfecta type II	*Bos taurus*	Fleckvieh, Red Angus, Holstein	*COL1A1*	AD	Small indel, missense	002127–9913	[10, 72, 73]
Disorders of bone mineralisation	Hypophosphatemic rickets	*Ovis aries*	Corriedale	*DMP1*	AR	Nonsense	001542–9940	[142]
	Hypophosphatasia^#^	*Ovis aries*	-	*ALPL*	AR	Missense	002162–9940	[143]
	Osteopetrosis with gingival hamartomas	*Bos taurus*	Belgian Blue	*CLCN7*	AR	Missense	001887–9913	[144]
Sulfation disorders	Osteopetrosis	*Bos taurus*	Red Angus	*SLC4A2*	AR	Large deletion	002443–9913	[145]
	Complex vertebral malformation	*Bos taurus*	Holstein	*SLC35A3*	AR	Missense	001340–9913	[146]
	Chondrodysplasia	*Ovis aries*	Texel	*SLC13A1*	AR	Small deletion	001400–9940	[147]
Filamins and related disorders	Skeletal-cardio-enteric dysplasia	*Bos taurus*	Romagnola	*MAP2K2*	AD	Missense	002381–9913	[148]
Primordial dwarfism and slender bones group	Arachnomelia	*Bos taurus*	Fleckvieh	*MOCS1*	AR	Frameshift deletion	001541–9913	[149]
	Arachnomelia	*Bos taurus*	Brown Swiss	*SUOX*	AR	Frameshift insertion	000059–9913	[150]
	Caudal vertebral scoliosis (Crooked tail)	*Bos taurus*	Belgian Blue	*MRC2*	AR	Frameshift deletion	001452–9913	[151]
	Primordial dwarfism	*Bos taurus*	Angus	*PRKG2*	AR	Nonsense	001485–9913	[152]
	Primordial disproportionate dwarfism	*Bos taurus*	Fleckvieh	*GON4L*	AR	Frameshift deletion	001985–9913	[153]
	Lethal multi-organ developmental dysplasia (paunch calf syndrome)	*Bos taurus*	Romagnola, Marchigiana	*KDM2B*	AR	Missense	001722–9913	[154, 155]
	Caprine-like generalized hypoplasia syndrome	*Bos taurus*	Montbeliarde	*CEP250*	AR	Nonsense	001502–9913	[156]
Proteoglycan core proteins disorders	Spondyloepimetaphyseal dysplasia	*Bos taurus*	Dexter, Scottish Highland	*ACAN*	AR	Frameshift insertion, regulatory	001271–9913	[157]
Chondrodysplasia punctata (CDP) group	Rhizomelic chondrodysplasia punctata	*Bos taurus*	Aubrac	*GNPAT*	AR	Splicing	002958–9913	[57]
Pseudoachondroplasia and the multiple epiphyseal dysplasias	Achondroplasia	*Ovis aries*	Cheviot	*PRICKLE1*	AR	Small deletion	002693–9940	[158]
Skeletal disorders caused by abnormalities of cilia or ciliary signaling	Ellis-van Creveld Syndrome	*Bos taurus*	Tyrolean Grey, Japanese Brown	*EVC2*	AR	Frameshift deletion, splice site, frameshift insertion	002540–9913	[159, 160]
Spondyloepi(meta)physeal dysplasias (SE(M)D)	Hereditary perinatal weak calf syndrome	*Bos taurus*	Japanese Black	*IARS*	AR	Missense	001817–9913	[161]
Polydactyly-Syndactyly-Triphalangism	Syndactyly	*Bos taurus*	Simmental Charolais cross, Simmental, Angus, Holstein	*LRP4*	AR	Missense, splice site, indel	000963–9913	[112–114]
Limb hypoplasia – reduction defects group	Tetradysmelia	*Bos taurus*	Holstein	*RSPO2*	AR	Frameshift deletion	002297–9913	[162]
	Tibial hemimelia syndrome	*Bos taurus*	Shorthorn, Galloway	*ALX4*	AR	Large deletion, duplication	001009–9913	[163]
Overgrowth (tall stature) syndromes and segmental overgrowth	Marfan syndrome	*Bos taurus*	Limousin, Japanese Black	*FBN1*	AD	Missense, splice site	000628–9913	[164]
	Skeletal dysplasia, disproportional tall stature	*Bos taurus*	Japanese Black, Mishima	*FGD3*	AID	Small deletion insertion	002625–9913	[165]
	Long tail	*Ovis aries*	Merinolandschaf	*HOXB13*	AD	Large insertion	002721–9940	[166]
Genetic inflammatory or rheumatoid-like osteoarthropathies	Dwarfism with inflammatory lesions	*Bos taurus*	Belgian Blue	*RNF11*	AR	Splice site	001686–9913	[167]
Dysplasias with multiple joint dislocations	Arthrogryposis multiplex congenita	*Bos taurus*	Red Dane	*CHRNB1*	AR	Small deletion	002022–9913	[168]
	Arthrogryposis multiplex congenita	*Bos taurus*	Angus	*AGRN*	AR	Large deletion	002135–9913	[143]
	Arthrogryposis, lethal syndrome	*Bos taurus*	Belgium Blue	*PIGH*	AR	Splice site	001953–9913	[169]
	Arthrogryposis, distal type 1B	*Bos taurus*	Holstein	*MYBPC1*	AD	Missense	001978–9913	[170]
Syndromes featuring craniosynostosis	Facial dysplasia syndrome	*Bos taurus*	Holstein	*FGFR2*	AD	Missense	002090–9913	[171]
	Frontonasal dysplasia	*Bos taurus*	Limousin	*ZIC2*	AD	Frameshift deletion	002307–9913	[172]
	Otocephaly	*Ovis aries*	Istrian Pramenka	*OTX2*	AD	Nonsense	002227–9940	[173]
Craniofacial Dysostoses	Hemifacial microsomia	*Bos taurus*	Romagnola	*LAMB1*	AR	Missense	002479–9913	[174]
	Cleft palate	*Bos taurus*	Limousin	*MYH3*	AR	Small deletion	002590–9913	[175, 176]
	Mandibulofacial dysostosis	*Bos taurus*	Hereford	*CYP26C1*	AR	Missense	002288–9913	[177]
	Brachygnathia	*Bos taurus*	Brown Swiss	*WNT10B*	AR	Duplication	002759–9913	[178]
	Brachygnathia, cardiomegaly and renal hypoplasia syndrome	*Ovis aries*	Merino	*OBSL1*	AR	Small deletions	001595–9940	[179]
Agenesis, hypoplasia or splitting of the bony core of the horn with or without skull malformations	Polled and multisystemic syndrome	*Bos taurus*	Charolais, Fleckvieh	*ZEB2*	AD	Frameshift deletion, large deletion	001736–9913	[180, 181]
	Scurs, type 2	*Bos taurus*	Charolais	*TWIST1*	AD	Duplication	001593–9913	[182]
	Polled/Horns, Celtic	*Bos taurus*	Angus, Galloway	*POLLED*	AD	Complex rearrangement	000483–9913	[183, 184]
	Polled/Horns, Friesian	*Bos taurus*	Holstein	*POLLED*	AD	Duplication	000483–9913	[185]
	Polled/Horns, Mongolian	*Bos taurus*	Kazakh	*POLLED*	AD	Complex rearrangement	000483–9913	[186]
	Polled/Horns, Guarani	*Bos taurus*	Nelore	*POLLED*	AD	Duplication	000483–9913	[187]
	Polled	*Ovis aries*	across breeds	*RXFP2*	AD	Insertion, large	000483–9940	[188]
	Polyceraty	*Ovis aries*	Damara, Jacob, Navajo-Churro, Sishui	*HOXD1*	AcD	deletion, small	000806–9940	[189]
Vertebral and costal dysostoses	Brachyspina	*Bos taurus*	Holstein	*FANCI*	AR	Large deletion	000151–9913	[190]
	Vertebral and spinal dysplasia	*Bos taurus*	Holstein	*TBXT*	AD	Missense	001951–9913	[191]

*MOI* Mode of inheritance, *AR* autosomal recessive, *AcD* autosomal co-dominant, *AD* autosomal dominant, *AID* autosomal incomplete dominant, *XLD* X-linked dominant

^*^phenotype categorization was adopted by the nosology of human genetic skeletal disorders[19]

^**#**^, *this entry is describing a genetically-modified organism (GMO)

The aims of our study were (1) to propose a nosology of the genetic skeletal disorders in cattle and sheep and (2) to contribute to the nosology with a number of novel genomically characterized cases.

## Methods

### Ethics statement

This multicentre study did not require regulatory or institutional ethical approval, as it was not experimental. The cases were submitted by owners or veterinary practitioners for diagnostic and surveillance purposes to monitor inherited disorders in cattle and sheep.

### Nosology of skeletal disorders in cattle and sheep

With the aim of collecting skeletal disorders previously reported in the literature, a Google Scholar and PubMed search was conducted using the terms *skeletal* and *genetic* or *horn,* and *sheep* or *cattle* in the ‘Title/Abstract’ field. The articles of potential interest were manually inspected and only those related to skeletal genetic disorders or horn traits with a known molecular cause were selected for a more detailed analysis. We also searched the OMIA database for *sheep* and *cattle disorders* with a known molecular cause and the phene category’skeleton phene’. The nosology of genetic skeletal disorders in cattle and sheep with a known molecular cause was then proposed with a disorder categorization, mode of inheritance, associated genes, and pathogenic variants following the example of the nosology of human genetic skeletal disorders [19].

### Animals and clinicopathological investigation

One or more cases were investigated for thirty bovine and nine ovine congenital forms of skeletal disorders. All parents were reported to be unaffected for the respective phenotypes, and maternal and paternal samples were also collected when available. Further details regarding the animals, phenotypes, and sample collection for each skeletal disorder can be found in Additional file 1, Figures S1 to S20. A proposal for a nosology of the described genetic skeletal disorders has been formulated in accordance with the nosology of human genetic skeletal disorders [19].

### DNA extraction

Genomic DNA was extracted from either ear cartilage or EDTA-stabilized blood from all affected animals and their parents when available (EDTA-stabilized blood from dams and sires or semen from bulls used in artificial insemination) using the Maxwell RSC DNA System (Promega).

### Whole-genome sequencing and variant calling

WGS data were generated for 35 selected cases (See Additional file 2, Table S1) and available parents using the Illumina NovaSeq6000 (Illumina Inc.) after preparation of Illumina TruSeq PCR-free fragment library with 2 × 150 bp paired-end reads at an average read depth of 20 × . The WGS data were generated based on the availability of samples, which were grouped into the following categories: complete trios, paternal halfsiblings, multiple cases and isolated single cases.

For the bovine cases, sequenced reads were aligned to the ARS-UCD1.2 reference genome [20]. Single-nucleotide variants (SNVs) and small indels were called as previously reported [21]. The applied software and steps to process fastq files into binary alignment map and genomic variant call format files followed the 1000 Bull Genomes Project (run 9) [22], except for trimming, which was performed using fastp [23]. Further processing of the genomic data was performed as previously reported [21]. To find private variants, we compared the genotypes of the bovine case(s) with 1037 bovine genomes of different breeds sequenced as part of the ongoing Swiss Comparative Bovine Resequencing Project (European Nucleotide Archive accession numbers PRJEB18113 and PRJEB83441).

For the ovine cases, the sequenced reads were aligned to the ARS-UI_Ramb_v2.0 (GCF_016772045.1) assembly [24] and the SNVs were called as described before, including the prediction of functional effects, in accordance with the best practices pipeline established in the Genome Analysis Toolkit [25]. To find candidate variants, the genotypes of the ovine case(s) were compared with those of 112 publicly available ovine genomes of unrelated breeds from the Swiss Comparative Ovine Resequencing Project (European Nucleotide Archive accession number PRJEB30931).

The presumed parentage based on pedigree records of all sequenced animals has been confirmed using identity-by-descent as implemented in the PLINK v1.9 software [26].

### Mode of inheritance, candidate gene and variant definition

Regarding the mode of inheritance (MOI), two different scenarios were hypothesized: (i) autosomal recessive MOI or (ii) dominant MOI, with each case considered as an isolated event, for example, due to a spontaneous de novo mutation. Subsequently, variant filtering was conducted for all cases based on the aforementioned two potential scenarios. In the event of a simple recessive MOI, the WGS data were filtered for homozygous private variants that were present exclusively in the case(s) and absent in this state in individuals of the control cohort. In the assumption of a recessive MOI with compound heterozygosity, WGS data were filtered for heterozygous variants that were present simultaneously only in the case(s) and absent in this state and in homozygosity in controls. Assuming a dominant MOI, the WGS data were filtered for heterozygous private variants that were present exclusively in the case(s) and absent in the controls. Subsequently, in the trio- and single-parent cohorts, variants were further prioritized based on the expected co-segregation of parental genotypes. Integrative Genomics Viewer (IGV) [27] was used for visual assessment of genomic regions containing potential candidate genes in all cases.

The term "candidate gene" is used to describe genes based on their known function and/or associated skeletal-related phenotypes in mammalian species. The term "candidate variant" is used to describe a variant deemed plausible considering the affected gene function and/or associated phenotype in mammalian species, rarity, and the predicted effect of the variant on the encoded protein, with at least two in silico tools predicting it to be deleterious. The variants were finally classified in accordance with the domestic animal variant classification guidelines [28]. All sequence accession numbers used for the candidate variants are listed in Additional file 2, Table S1.

### Occurrence of variants in global control cohorts

For the bovine cases, the comprehensive variant catalogue from run 9 of the 1000 Bull Genomes Project was available for investigation of the distribution of variants within a global control cohort [22]. The complete dataset includes 5,116 bovine genomes, including 576 from the Swiss Comparative Bovine Resequencing Project, representing a diverse range of over 130 breeds.

For the ovine cases, the comprehensive variant catalogue from the Sheep Genomes Project Variant Database was available for investigation of the distribution of variants within a global control cohort [29]. The complete dataset includes 935 ovine genomes from 62 distinct breeds, accessible via the CSIRO Data Access Portal [30].

### Variant prioritization and in silico assessment of the molecular consequences

The VarElect v5.23 software (LifeMap Sciences Inc) [31] was used for variant selection for phenotype-dependent gene variant prioritisation according to the phenotype query "skeletal OR bone" to identify variants in possible candidate genes.

PredictSNP1 [32], PolyPhen-2 [33], SIFT [34], SNAP [35], MAPP [36], PhD-SNP [37] and/or MutPred2 [38] were used to predict the biological consequences of the candidate protein-changing variants. A variant was considered to be deleterious if there was a prediction that the variant would be deleterious with the use of two or more of the above tools. The gnomAD browser was used to evaluate the tolerance to loss-of-function mutations of the orthologous human gene (pLI score) [39]. ESE motif prediction was performed using ESEfinder 3.0 software in the context of the *PPIB* variant to identify the creation or disruption of putative splicing regulatory elements [40].

### Targeted genotyping for variant validation

DNA samples from the common sire and four further ovine cases of *COL2A1*-related achondrogenesis, type II (cases 4–7), the common sire and five unaffected half sibs of the ovine cases of *COL1A1*-related osteogenesis imperfecta (cases 11–13), and the sire and dam of the bovine case 29 affected by syndactylism were used for targeted genotyping of the respective candidate variants.

PCR and subsequent Sanger sequencing was performed using the following primers respectively: *COL2A1*_Forward 5´-GGAGTACTGACCTGACCCC-´3 and *COL2A1*_Reverse 5´-CGTCCCCACTTACCGAGG-´3; *COL1A1*_Forward 5´-CCCATGCTCCTCCCTAACTC-´3 and *COL1A1*_Reverse 5´-ACAAATTGAGCCCAGGAGTG-´3; *CD4*_Forward 5´-AGGGCTCTCTTCTGTAAACAGGG-´3 and *CD4*_Reverse 5´-CCTCTCTTAGGCACCTGTTCTTGG-´3.

After amplification with AmpliTaqGold360Mastermix (Thermo Fisher Scientific), the purified PCR amplicons were directly sequenced on an ABI3730 capillary sequencer (Thermo Fisher Scientific) and the results analyzed using Sequencher 5.1 software (GeneCodes).

### Investigation of copy number variation

To assess possible larger structural variants and chromosomal abnormalities, including numerical and structural abnormalities, the depth of coverage across all chromosomes was calculated. A sliding window approach was used with a 10 kb and 200 kb window sizes. The number of reads within each specified window was determined using the bedcov function of Samtools [41]. Coverage plots were generated using the Manhattan function of the R package qqman [42].

### Comparative chromosomal alignment

The Comparative Genome Viewer [43] was used to compare two genomes based on assembly-assembly alignments provided by National Center for Biotechnology Information (NCBI), for bovine chromosomes 23 and 28, between the *Bos taurus* assembly ARS-UCD2.0 (GCF_002263795.3) and the *Homo sapiens* assembly GRCh38.p14 (GCF_000001405.40).

## Results and discussion

### Nosology of skeletal disorders in cattle and sheep

The proposed nosology of skeletal disorders in cattle and sheep for the previously reported skeletal disorders with a known molecular cause is summarized in Table 1. These represent 43 different disorders caused by variants in 45 genes, which were grouped into 21 categories adapted from the human medical nosology: *FGFR3*-related chondrodysplasias, type 2 collagen disorders, osteogenesis imperfecta and bone fragility group, disorders of bone mineralization, sulfation disorders, filamins and related disorders, primordial dwarfism and slender bones group, chondrodysplasia punctata (CDP) group, proteoglycan core proteins disorders, skeletal disorders caused by abnormalities of cilia or ciliary signaling, spondyloepi(meta)physeal dysplasias (SE(M)D), acromesomelic dysplasias, polydactyly-syndactyly-triphalangism, limb hypoplasia – reduction defects group, overgrowth (tall stature) syndromes and segmental overgrowth, genetic inflammatory or rheumatoid-like osteoarthropathies, dysplasias with multiple joint dislocations, syndromes featuring craniosynostosis, craniofacial dysostoses, agenesis, hypoplasia or splitting of the bony core of the horn with or without skull malformations, and vertebral and costal dysostoses.

### Novel genomically characterized cases

The 39 cases were associated with 19 different congenital skeletal disorders (Table 2). These 19 disorders were further grouped in nine categories based on the human nosology [19] considering the clinicopathological and genetic findings: type 2 collagen disorders, osteogenesis imperfecta and bone fragility group, osteopetrosis and related osteoclast disorders, sulfation disorders, primordial dwarfism and slender bones group, polydactylism-syndactylism-triphalangism group, disorders of bone mineralisation, dysplasias with multiple joint dislocations, and vertebral and costal dysostoses. For six disorders belonging to the four different categories (primordial dwarfism and slender bones group, disorders of bone mineralization group, dysplasias with multiple joint dislocations group, and vertebral and costal dysostoses group) no molecular cause could be identified (Table 2). Twelve disorders were associated with 21 SNVs or small indels. Among these, 17 candidate variants were identified, affecting 16 distinct genes. Of these, 11 were classified as pathogenic and six as likely pathogenic. Additionally, four variants were categorized as being of uncertain significance. Furthermore, a trisomy and partial monosomy were the cause of two different disorders. The overall phenotypical classification, associated genes and variants, and hypothesised MOI are presented in Table 2.

**Table 2:** The nosology of the genetic skeletal disorders in the 39 cases studied: categorization, hypothesized mode of inheritance, associated genes and potential causal variants

Category*	Skeletal disorder	Species	Breed	Gene	MOI	Variant type	Variant classification^#^	Cases ID
Type 2 collagen disorders	Achondrogenesis, type II	*Bos taurus*	Holstein	*COL2A1*	AD	Heterozygous, small frameshift deletion	Pathogenic	1
	Achondrogenesis, type II	*Ovis aries*	Crossbred	*COL2A1*	AD	Heterozygous, missense	Pathogenic	2–7
Osteogenesis imperfecta and bone fragility group	Osteogenesis imperfecta	*Bos taurus*	Holstein	*PPIB*	AR	Homozygous, intronic	Uncertain significance	8
	Osteogenesis imperfecta	*Bos taurus*	Stabiliser	*COL1A2*	AD	Heterozygous, missense	Pathogenic	9–10
	Osteogenesis imperfecta	*Ovis aries*	Crossbred	*COL1A1*	AD	Heterozygous, missense	Pathogenic	11–13
Osteopetrosis and related osteoclast disorders	Osteopetrosis and brachignatia inferior	*Bos taurus*	Crossbred	LOC112445140	XLD	Heterozygous, stop gained	Pathogenic	14
Sulfation disorders	Caudal and thoracic vertebral and viscerocranial malformations	*Bos taurus*	Holstein	*SLC40A1*	AD	Heterozygous, missense	Pathogenic	15
Primordial dwarfism and slender bones group	Primordial proportionate dwarfism	*Bos taurus*	Simmental	–	–	–	–	16
	Primordial proportionate dwarfism	*Bos taurus*	Simmental	*PTPN9*	AD	Heterozygous, missense	Uncertain significance	17
	Primordial disproportionate dwarfism	*Bos taurus*	Angus	*PRDM10*	AD	Heterozygous, missense	Pathogenic	18–19
	Primordial disproportionate dwarfism	*Bos taurus*	Holstein	–	–	–	–	20
	Primordial disproportionate dwarfism with craniofacial dysmorphism	*Bos taurus*	Holstein	*PDGFRA*	AD	Heterozygous, missense	Pathogenic	21
	Primordial disproportionate dwarfism	*Bos taurus*	Crossbred	*ABCC8*	AD	Heterozygous, missense	Likely pathogenic	22
	Arachnomelia	*Bos taurus*	Holstein	–	–			23
	Craniofacial dysmorphism-hydrocephalus-dwarfism syndrome	*Bos taurus*	Angus	Trisomy 23	AD	Aneuploidy	Pathogenic	24
Polydactyly-Syndactyly-Triphalangism	Polydactyly	*Bos taurus*	Crossbred	Partial monosomy chr28	AD	Aneuploidy	Pathogenic	25
	Syndactyly	*Bos taurus*	Holstein	*LRP4*	AR	Compound heterozygous, small frameshift insertion, missense	Pathogenic	26
	Syndactyly	*Bos taurus*	Holstein	*LRP4*	AR	Compound heterozygous, small frameshift insertion, missense	Pathogenic	27
	Syndactyly	*Bos taurus*	Holstein	*KMT2C*	AR	Homozygous, missense	Uncertain significance	28
	Syndactyly	*Bos taurus*	Droughtmaster	*CD4*	AR	Homozygous, missense	Uncertain significance	29
Disorders of bone mineralisation	Congenital rickets	*Bos taurus*	Angus	–	–	–		30–31
Dysplasias with multiple joint dislocations	Craniofacial dysmorphisms, and forelimbs skeletal dysplasia	*Bos taurus*	Holstein	*CCT3*	AD	Heterozygous, missense	Likely pathogenic	32
	Craniofacial dysmorphisms, forelimbs dislocations and skeletal dysplasia	*Bos taurus*	Holstein	*ITGAE*	AR	Homozygous, missense	Likely pathogenic	33
	Craniofacial dysmorphism, forelimbs dysplasia with joint contracture	*Bos taurus*	Chianina	*CNTNAP1*	AR	Compound heterozygous, missense, intronic	Likely pathogenic	34
	Forelimbs dysplasia with joint contracture	*Bos taurus*	Holstein	*NFE2L1*	AD	Heterozygous, small disruptive inframe deletion	Likely pathogenic	35
	Forelimbs dysplasia with joint contracture	*Bos taurus*	Chianina	*IL16*	AD	Heterozygous, missense	Likely pathogenic	36
	Forelimbs dysplasia with joint contracture	*Bos taurus*	Limousin	–	–	–	–	37–38
Vertebral and costal dysostoses	Hemifacial microsomia with hemivertebrae	*Bos taurus*	Rendena	–	–	–	–	39

*MOI* Mode of inheritance, *ID* identification number, *AR* autosomal recessive, *AD* autosomal dominant, *XLD* X-linked dominant

^*^phenotype categorization was adopted by the nosology of human genetic skeletal disorders [19]

^**#**^variants were classified according to the animal variant classification guidelines to objectively evaluate genetic variant pathogenicity in domestic animals [28]

WGS was performed for 35 of the 39 cases; the remaining 4 were identically affected paternal halfsiblings. A WGS trio-approach was carried out in 14 cases, a WGS single-parent approach in 7 cases with no access to one of the parents, a WGS with paternal half-siblings-approach in 4 cases and a WGS single-case approach in 10 cases. An overall genetic diagnosis associated with the presence of a likely pathogenic or pathogenic variant including aneuploidies was achieved in 64% of cases, specifically 58% with the trio-approach, 75% with the paternal halfsibling approach, and 75% with multiple cases approach and 70% with a single-case approach (Table 2; see Additional file 2 Table S1). The efficiency of WGS for genetic diagnosis in cattle was recently investigated for two lethal congenital syndromes, achieving diagnostic rates ranging from 33 to 50% [44, 45]. The overall results of the current study show a higher diagnostic rate compared to the genomic studies of schistosoma reflexum and congenital syndromic Chiari-like malformation in cattle [44, 45]. They are also promising when compared to the efficacy of WGS-based genetic diagnosis in humans, where putative causal genetic variants have been reported in 25% of patients [46].

Heterozygous, probably dominant, candidate causal variants were found in 23 cases in five disorder categories, while candidate causal variants for recessive alleles were described in seven cases in four disease categories. In addition to these autosomal variants, a single heterozygous bovine X-linked candidate causal variant was found in a single female case (Table 2, see Additional file 2 Table S1). The identified likely dominant acting variants either arose post-zygotically in the developing embryos or were inherited from a germinal mosaic parent as shown below. Unfortunately, it was not possible to clarify the origin of the identified variants in individual cases where samples were not available from both parents.

Two cases showed paternally inherited aneuploidies: trisomy 23 and partial monosomy 28 (Table 2, see Additional file 2 Table S1). A recent, comprehensive study on screening for interchromosomal rearrangements compatible with normal spermatogenesis in cattle demonstrated that, in this species, aneuploidies are more frequently due to defective paternal meiosis [47], as was observed in our two cases.

The following subsections present a comprehensive overview of the genomic findings of genetic skeletal disorders within seven distinct nosological categories, for which candidate causal variants were identified and a summary of cases with an unidentified genetic cause. The clinicopathological details can be found in Additional file 1 Figures S1 to S20 and further genomic description can be found in Additional file 2 Table S1.

### Type 2 collagen disorders

**Fig. 1:**
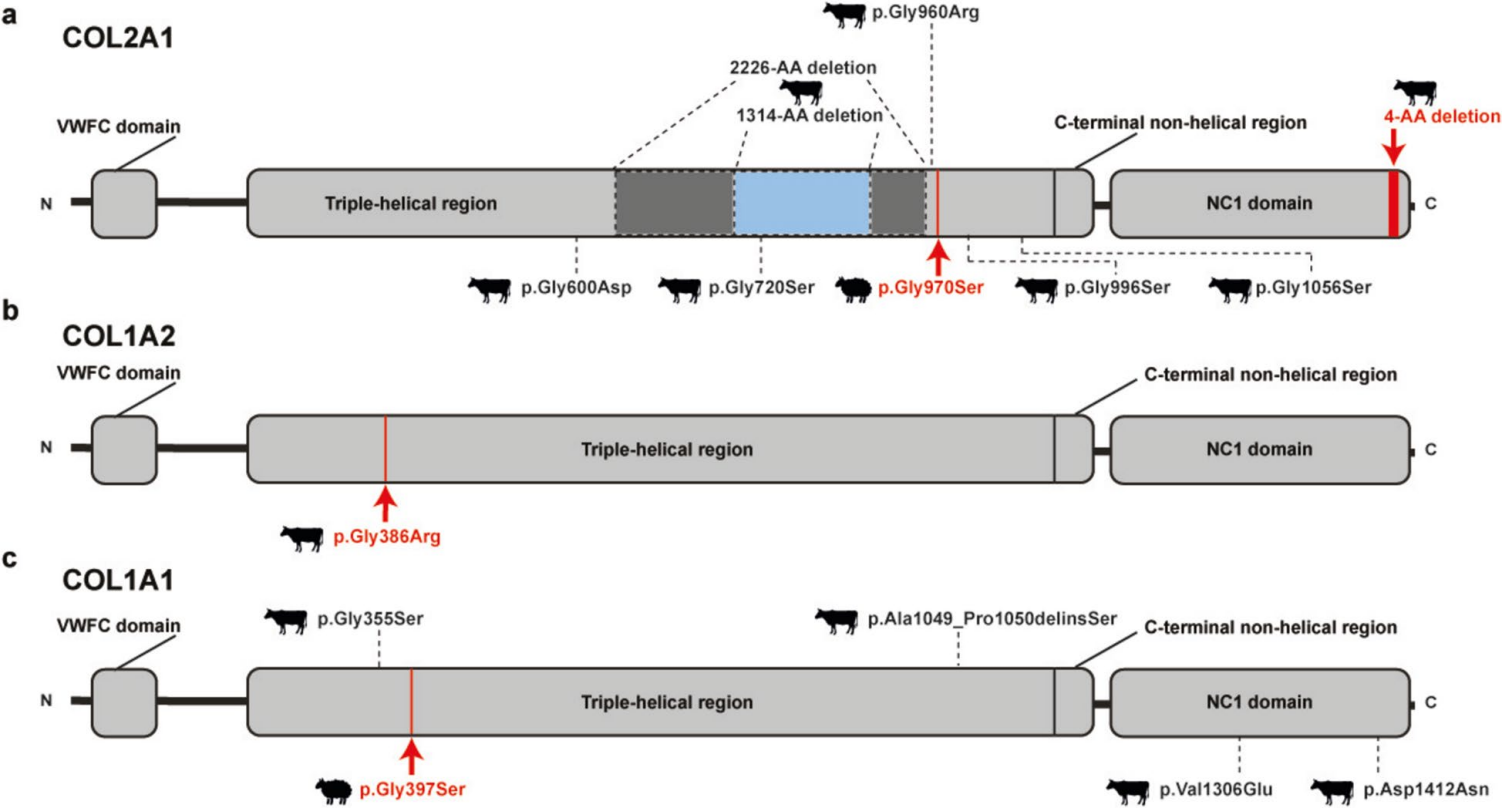
*COL2A1*, *COL1A1* and *COL1A1* skeletal disorder-associated variants in cattle and sheep. **a** Schematic representation of the COL2A1 protein. The previously reported variants associated with achondrogenesis type II are indicated with dotted lines and the newly identified variants in cattle and sheep are indicated by red arrows. The novel identified variants include: 4-AA deletion in an isolated Holstein calf (case 1) and p.Gly970Ser in crossbred lambs (cases 2–7). The previously reported cases include: p.Gly600Asp in Charolais x Salers crossbred calves[10], p.Gly720Ser in an isolated German Holstein calf [10], p.Gly960Arg in French Holstein calves [54], p.Gly996Ser in German Holstein calves [10] and p.Gly1056Ser in an isolated Holstein calf [50], a 6672-bp deletion in an isolated Italian Holstein calf [52], and a 3943-bp deletion in an isolated Danish Holstein calf [139]. **b** Schematic representation of the COL1A2 protein. The novel identified variant (p.Gly386Arg) in Stabiliser calves (cases 9 and 10) associated with osteogenesis imperfecta (OI) type II is indicated by a red arrow. **c** Schematic representation of the COL1A1 protein. The previously reported variants associated with OI are indicated with dotted lines and the newly identified variant (p.Gly397Ser) in crossbred lambs (cases 11–13) is indicated with a red arrow. The previously reported cases include: p.Gly355Ser in Red Angus calves [72], p.Ala1049_Pro1050delinsSer in Simmental calves [10], p.Val1306 in an isolated Holstein calf and p.Asp1412Asn in Normande calves [74]

A single bovine case with both unaffected parents (case 1, trio approach) and a series of ovine cases with a common sire (cases 2–7, single-parent approach) were diagnosed with achondrogenesis type II (Fig. 1). Both phenotypes were classified as type 2 collagen disorders according to the proposed nosology.

#### Achondrogenesis type II in cattle and sheep

A Holstein calf (case 1) was clinically diagnosed with a mild form of achondrogenesis. The SNVs and small indel prioritization approach applied to case 1 did not identify a single possible candidate variant in the bovine case. Therefore, in view of the presented phenotype, we visually inspected the candidate gene *COL2A1* on IGV. With this approach, a heterozygous pathogenic 12 bp deletion affecting exon 54 was identified, exclusively present in case 1 and absent in both parents and controls. The heterozygous *COL2A1* variant (Chr5:g.32312706_32312718del; c.4432_4443del; p.(Gly1478_Ile1481del)) leads to an in-frame deletion of four amino acids, presumably affecting the C-terminal end of the protein (Fig. 1a). We believe that the variant arose de novo in the developing embryo, as we found no variant read in the sequenced genome data from either parent, or was inherited from the sire with a very low level of mosaicism.

In sheep, six paternal half-sib crossbred lambs were aborted (cases 2–7), all progeny of the same Charollais ram. The pathological presentation resembled the *COL2A1*-related achondrogenesis type II previously reported in cattle [48]. WGS of two ovine cases was obtained and assuming a dominant MOI, a single shared candidate pathogenic heterozygous missense variant in exon 43 of ovine *COL2A1* was identified (Chr3:g.138610131G > A; c.2908G > A; p.Gly970Ser). The exchanged amino acid of COL2A1 is located in the triple-helical region (Fig. 1a). The variant was further genotyped using PCR and Sanger sequencing in all six cases and in the common sire. We hypothesize that the variant was inherited from the mosaic sire, as all the affected half sibs were confirmed heterozygous, and a low proportion of the variant allele was detected in semen DNA but not in blood DNA from the sire.

In both bovine and ovine cases, the identification of novel variants affecting *COL2A1* confirmed the clinicopathological diagnoses. Here we report the first cases of ovine *COL2A1*-related achondrogenesis type II and the first bovine *COL2A1*-related case that survived after parturition. The disease phenotype in sheep was very similar to that previously reported in bovine *COL2A1*-related achondrogenesis type II (OMIA:001926-9913). In the bovine case 1, the overall bone malformations observed were less severe than previously reported [48], possibly due to location of the variant almost at the C-terminal end of the COL2A1 protein. For many genes it is known that the type of genetic alteration influences the phenotypic outcome, e.g. the severity of a congenital defect varies or is completely different depending on the individual variant [49]. However, to date, with the exception of case 1, a uniform phenotype has been observed for *COL2A1*-related achondrogenesis type II in cattle, irrespective of the variant type.

A total of eight independent pathogenic dominant variants in *COL2A1* have been previously identified in cattle and are thought to be responsible for achondrogenesis type II associated with abortion and severe malformations (Fig. 1a) [10, 48, 50–54]. Two large deletions in the triple-helical region have been identified and predicted to result in non-expression of the variant allele in cattle [48, 52]. The less severe phenotype observed in case 1 could be due to reduced expression of the encoded protein, rather than a complete absence of expression, as was previously hypothesized in cases where a frameshift variant were identified. In addition, the in-frame deletion identified in our study affects the end of the fibrillar collagen NC1 domain, in contrast to the previously reported variants that disrupt the triple helical region. The Gly-X-Y structural motif is essential for the assembly of the collagen triple helix [10, 50, 51, 54]. In the ovine cases reported here, the identified variant also disrupts the Gly-X-Y structural motif. With the presented cases, we extend the number of affected species and the phenotypic presentation of *COL2A1*-related achondrogenesis type II, highlighting that the disease is present in sheep and that affected animals can survive after birth.

### Osteogenesis imperfecta and bone fragility group

Three bovine cases (cases 8–10, trio approach) and three ovine cases with a common sire each (cases 11–13, single-parent approach) were diagnosed with osteogenesis imperfecta (OI). All phenotypes were classified as disorders belonging to the osteogenesis imperfecta and bone fragility group, in accordance with the proposed nosology of genetic skeletal disorders.

#### Osteogenesis imperfecta in cattle and sheep

A stillborn female Holstein calf (case 8) was pathologically diagnosed with OI and dentinogenesis imperfecta. Assuming a recessive MOI, the WGS-trio approach enabled the identification of a homozygous variant that is of uncertain significance in intron 3 upstream of exon 4 of *PPIB* (Chr10:g.45822912G > C; c.320-443G > C). *PPIB* is associated with a perinatal lethal recessively inherited form of OI type IX in humans (OMIM:259440), where affected individuals present with short stature, congenital fractures, scoliosis, kyphosis, and white to gray sclerae [55]. Although the majority of the variants associated with this condition in humans are located within coding exons, we hypothesized that the identified variant may represent an intronic variant that could result in a reduction or absence of PPIB protein expression. Several Mendelian disorders have been associated with intronic variants [56, 57]. To substantiate our hypothesis, further research is required to provide additional insights into the biological effects of the identified *PPIB* variant. Unfortunately, no RNA samples were available in the current study to investigate potential different transcripts. Additional similar Holstein cases could be tested for the identified variant in *PPIB* and in case of occurrence, material for RNA experiments could be collected for validation.

Two full-term paternal half-sib Stabiliser calves (cases 9 and 10) were aborted and pathologically diagnosed with OI. On the assumption of a recessive MOI, no homozygous candidate variants were identified. Conversely, assuming a dominant MOI, a single pathogenic heterozygous missense variant in exon 21 of *COL1A2*, located in the triple-helical region (Chr4:g.11792118G > A; c.1156G > A; p.Gly386Arg), was identified. This missense variant is predicted to result in a substitution of a glycine residue and is predicted to disrupt the Gly-X-Y structural motif, which is essential for the assembly of the collagen triple-helix (Fig. 1b). The variant may be a de novo mutation inherited from a germinal mosaic sire. In humans, *COL1A2* is associated with dominantly inherited forms of OI (types II to IV), with both dominant- and recessively inherited forms of Ehlers-Danlos syndrome (EDS), and with a combined form of OI and EDS (OMIM:120160) [58–61]. In particular, OI type II is a perinatal lethal form that shares similar findings to those observed in our bovine cases. This severe form is frequently associated with variants that result in the substitution of the glycine residue in almost any of the Gly-X–Y tripeptide motifs found in the triple helical region of the alpha chain [62]. This may reflect the possibility that different variants lead to lethality via different pathways [62–64]. Furthermore, three pathogenic variants in *COL1A2* have been found in lethal forms of OI in dogs (OMIA:002112-9615) [65–67]. Herein, we provide the first report of a *COL1A2*- related form of OI type II in cattle.

Three paternal half-sib crossbred lambs (cases 11–13), progeny of the same Charollais, were aborted and pathologically diagnosed with OI. Assuming a dominant MOI, a heterozygous missense variant in exon 18 of *COL1A1*, located at the triple-helical region (Chr11:g. 36197409G > A; c.1189G > A; p.Gly397Ser), was identified as a potential causal variant. Similarly, in this case series, the pathogenic variant results in a substitution of a glycine residue and is predicted to disrupt the essential Gly-X-Y structural motif for the assembly of the collagen triple-helix, ultimately leading to lethality (Fig. 1c). The *COL1A1* variant was experimentally identified as heterozygous in all three cases, the common ram, and absent in five unaffected half-sibs. We hypothesize that the variant was inherited from the mosaic sire.

Variants in *COL1A1* in humans are also associated with dominantly inherited forms of OI (types I to IV), with a dominantly inherited form of EDS, and with a combined form of OI and EDS (OMIM:120150) [68–70]. Additionally, a dominantly inherited variant in *COL1A1* causing OI type II has been described in dogs (OMIA:002126-9615) [71] and four in cattle (OMIA:002127-9913), three of which were inherited from a germinal mosaic sire [10, 72–74]. Despite previous reports of OI in sheep, the underlying cause has not been investigated [75]. Therefore, this is the first report of a *COL1A1*-related form of OI type II in sheep.

### Osteopetrosis and related osteoclast disorders

#### Syndromic osteopetrosis in cattle

A female Angus x Holstein crossbred calf (case 14) was pathologically diagnosed with a syndromic form of osteopetrosis. The trio-approach identified a heterozygous pathogenic stop-gained variant in exon 1 of LOC112445140, also known as DDB1- and CUL4-associated factor 12-like protein (ChrX: g.10872688G > T; c.332C > A; p.Ser111*). We think that the variant arose post-zygotically as a spontaneous de novo mutation during the development of the embryo, as no variant reads were observed in either parental DNA. In mice, the orthologous DDB1- and CUL4-associated factor 12-like gene is moderately expressed in the early conceptus, limbs and musculoskeletal system, as well as the intestine. Additionally, it displays high expressed in the cardiovascular system [76]. DDB1-CUL4 associated factors (DCAFs) are vital for optimal cellular function in diverse organ systems, with a particularly well-documented role in physiological bone growth and bone metabolism [77]. It is hypothesized that DCAFs act as substrate receptors, determining the specificity of the ubiquitination machinery through E3 ubiquitin ligases [77]. Abnormal expression or function of E3 ubiquitin ligases can result in bone disorders including osteoporosis, ankylosing spondylitis, and osteoarthritis [78, 79]. Considering the predicted deleterious effect of the identified variant in case 14, we speculate that the variant may disrupt the ubiquitination machinery and may be responsible for the observed phenotype if the LOC112445140 encodes a functional protein.

In humans, the *DCAF12L2* represents an intronless retrocopy of a related multi-exon gene located on chromosome 9 and it is known to be transcribed [80]. Based on 100% identity with the human DCAF12L2 protein sequence, the LOC112445140 has been considered an orthologous. Functional experiments would be required to confirm the causality of the identified variant. With this example, we highlight that syndromic skeletal genetic disorders might be associated with the DCAFs.

### Sulfation disorders

#### Caudal and thoracic vertebral and viscerocranial malformations in cattle

A Holstein heifer (case 15) was clinically diagnosed with scoliosis of the caudal vertebra (“crooked tail”), thoracic scoliosis, and skull dysplasia. The trio-approach identified a heterozygous missense variant in exon 5 of *SLC40A1*, affecting the ferroportin-1 domain of SLC40A1 (Chr2:g.6785954 T > A; c.323 T > A; p.Ile108Asn). The pathogenic variant most likely arose post-zygotically due to a spontaneous de novo mutation during the development of the embryo, as no variant reads were observed in either parental DNA. In humans, *SLC40A1* has been associated with dominantly inherited adult-onset hemochromatosis type 4 (OMIM:606069), a disorder with a heterogeneous phenotype including cataract, cardiomyopathy, hepatic fibrosis, joint pain and osteoarthritis, anemia, and elevated serum ferritin and transferrin saturation [81, 82]. In the bovine case 15, the clinical presentation was not compatible with hemochromatosis. However, neither cardiac and liver imaging studies nor hematological and biochemical analyses were performed. Therefore, a possible cardiopathy, hepatic fibrosis or anemia cannot be excluded. One similarity noted was the presence of a cataract. Interestingly, *SLC40A1* plays an important role in the transport of iron from the intracellular to the extracellular spaces, and disruption of this mechanism can induce cellular ferroptosis [83]. Ferroptosis is mainly involved in iron metabolism and lipid peroxidation. Iron overload is known to be strongly associated with cellular ferroptosis, and iron overload and lipid peroxide accumulation together lead to bone destruction and ultimately musculoskeletal diseases such as osteoporosis, osteoarthritis, intervertebral disc degeneration, sarcopenia, and rhabdomyolysis [83, 84]. In addition, heterozygous *Slc40a1* mutant mice show decrease bone mineral content and density [85]. However, to date, no congenital skeletal malformations have been associated with variants in *SLC40A1* in any mammalian species. Therefore, here we propose a possible novel candidate gene for similar congenital skeletal disorders.

### Primordial dwarfism and slender bones group

Eight bovine cases (trio-approach: cases 16, and 24; single cases: cases 17, 20–22; multiple cases: cases 18 and 19) were diagnosed with dwarfism and one bovine case (trio-approach: case 23) was diagnosed with arachnomelia. These disorders were considered to belong to the group of primordial dwarfism and slender bones of the proposed nosology. In two of them (cases 16 and 23), no plausible candidate variant was identified, which is discussed in more detail later.

#### Primordial proportionate dwarfism in cattle

A Simmental heifer (case 17) was clinically diagnosed with primordial proportionate dwarfism. Based on the assumption of a recessive MOI, no candidate variants have been identified as homozygous. Assuming a dominant MOI, a heterozygous uncertain significance missense variant in exon 7 of *PTPN9* has been identified and exchanges the encoded amino acid of PTPN9 at position 276, located in the protein-tyrosine phosphatase region (Chr21:g.33356490G > A; c.826G > A; p.Ala276Thr). The variant could be a de novo mutation, but the lack of parental DNA did not allow us to investigate this further. However, both parents were normal-sized. It is noteworthy that *Ptpn9* mutant homozygous mice show embryonic growth retardation, a reduction in body size, and an abnormal morphology of long bone diaphysis [86, 87]. In addition, *PTPN9* physically interacts with *GHR* that is associated with the dominantly inherited human growth hormone insensitivity (OMIM:604271), which is characterized by short stature [88, 89]. We hypothesize that a disruption in *PTPN9* might lead to a malfunction of the encoded protein, resulting in the observed phenotype observed in case 17. However, the identified variant in *PTPN9* is of uncertain significance, so a causal relationship with primordial dwarfism could not be established.

#### Primordial disproportionate dwarfism in cattle

Two half-sib Angus calves (cases 18 and 19) were pathologically diagnosed with chondrodysplasia leading to primordial disproportionate dwarfism. No shared homozygous variants were identified in the two genomes as candidates for a causal recessive allele. Assuming a dominant MOI, a heterozygous pathogenic missense variant was found in exon 6 of *PRDM10* leading to an amino acid exchange in PRDM10 at position 289, located in the PR-SET domain (Chr29:g.36138136G > A; c.866C > T; p.Pro289Leu). We speculate that the candidate variant was inherited from a germinal mosaic sire. Unfortunately, no parental samples were available to confirm this. *PRDM10* plays an important role in maintaining translation, in part mediated by EIF3B*,* and is essential for early embryonic development and embryonic stem cell homeostasis [90]. *PRDM10* is moderately expressed in bone and in heterozygous *Prdm10* mutant mice a decreased bone mineral content was observed [85]. Interestingly, according to Disease Novelty (TIN-X) from Pharos interface [91], *PRDM10* is predicted to be associated with congenital bone disorders, including osteochondrodysplasia and OI. In addition, EIF3B variants are predicted to cause Ikegawa type craniotubular dysplasia in humans, characterized by short stature, macrocephaly, dolichocephaly, or prominent forehead [92–94]. However, this is the first report of a bovine form of primordial disproportionate dwarfism that might be associated with *PRDM10*. In human, a dominantly inherited variant in *PRDM10* in a single family has been associated with Birt-Hogg-Dube syndrome 2 (OMIM620459), which is characterized by lipomatosis, fibrofolliculomas, and renal cell carcinoma, as well as other cancers [95]. Although the clinicopathological presentation differs from that of our bovine cases, considering the known function of *PRDM10*, its expression in bone, and the classification of the identified variant as pathogenic, supporting evidence suggests that the observed phenotype in the bovine cases might represent an expansion of the spectrum of *PRDM10*-related diseases.

A Holstein calf (case 21) was clinically diagnosis with primordial disproportionate dwarfism and craniofacial dysmorphism. In the absence of a homozygous candidate for a causal recessive allele, a dominant MOI was assumed. A heterozygous pathogenic missense variant in exon 12 of *PDGFRA,* which replaces residue 562, was found and might be due to a spontaneous de novo mutation. However, due to the lack of parental DNA, we could not prove this. The likely pathogenic variant is located in the PTKc PDGFR alpha domain (Chr6:g.69749162 T > C; c.1685 T > C; p.Ile562Thr) and is predicted to result in an altered transmembrane protein, loss of strand, and modified stability of the PDGFRA [38]. *PDGFRA* encodes a receptor tyrosinase kinase that is fundamental for normal embryo development and maintenance of homeostasis [96]. It plays an important role in cranial and skeletal morphogenesis of the embryo [97–99]. Moreover, it mediates the activation of the ERK MAPK pathway that if impaired can be associated with RASopathies, as previously mentioned [100, 101]. Indeed, *Pdgfra* homozygous mutant mice show craniofacial dysmorphisms, delayed embryonic growth, and skeletal dysplasia while *Pdgfra* heterozygous mutant mice display moderate skeletal defects affecting the ribs, vertebrae and sternum [102–105]. This report presents the first documented bovine form of primordial disproportionate dwarfism with craniofacial dysmorphism possibly associated with *PDGFRA*, expanding the knowledge of the phenotype-gene relationship.

A Limousin x Brown Swiss crossbred calf (case 22) was clinically diagnosed with primordial proportionate dwarfism. As expected, no homozygous candidate for a causal recessive allele was identified. However, assuming a dominant MOI, a heterozygous likely pathogenic missense variant in exon 24 of *ABCC8* was identified. This candidate variant of uncertain significance leads to an amino acid in the ATP-binding cassette transporter C region of ABCC8 (Chr15:g.35095352A > G; c.2875A > G; p.Met959Val). This could be a de novo mutation but lacking parental DNA we were unable to prove this. *ABCC8* is associated with a dominantly inherited form of hyperinsulinemic hypoglycemia in children, which may be inherited from a parent or arise de novo in the developing embryo [106]. Patients affected by this condition may present with seizures and hypoglycemia [106]. In the absence of data regarding insulin and glucose levels, it is not possible to ascertain whether the affected animal exhibited hyperinsulinemic hypoglycemia. It is nevertheless of interest to note that the Disease Novelty (TIN-X) from the Pharos interface predicts that *ABCC8* is involved in bone developmental disorders, including achondrodysplasia, osteochondrodysplasia, syndactyly, brachydactyly, multiple epiphyseal dysplasia, spondyloepiphyseal dysplasia, acrofacial dysostosis, and OI [91]. Moreover, *Abcc8* has been associated with dwarfism in rats [107]. Considering these findings, it seems reasonable to suggest that the identified variant in *ABCC8* may be responsible for the observed primordial proportionate dwarfism in case 22, representing the first spontaneous case of *ABCC8*-related primordial proportionate dwarfism.

#### Craniofacial dysplasia-hydrocephalus-dwarfism syndrome in cattle

**Fig. 2:**
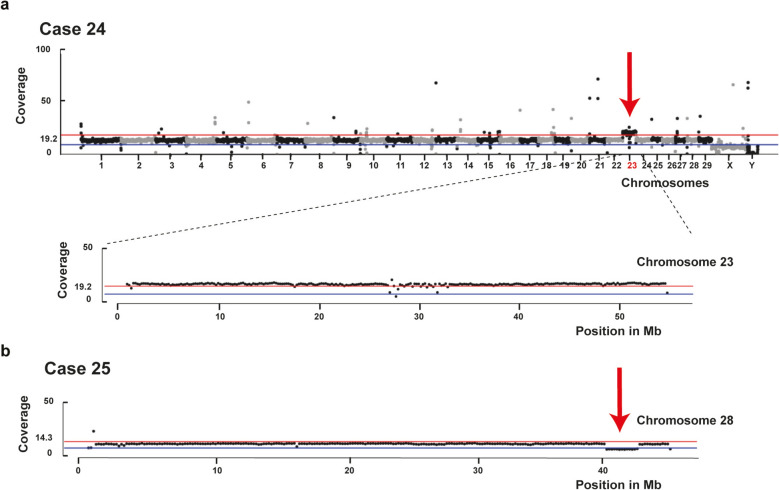
Structural variants associated with skeletal disorders in cattle. **a** Trisomy 23 in a crossbred calf with craniofacial dysmorphism-hydrocephalus-dwarfism syndrome (case 24). Coverage plot of all 29 autosomes as well as the X and Y chromosomes with a 200‐kb window of the affected calf. Note the extra copy of chromosome 23. Below, the coverage plot of chromosome 23 of the affected calf is presented. Note the increasing of the coverage across all chromosome 23 (trisomy). The average coverage across the genome is 19.2 × . Red line: mean genome‐wide average coverage. Blue line: 0.5 × mean coverage as observed in the haploid coverage of X chromosome as expected in a male animal. **b** Chromosome 28 partial monosomy in a crossbred calf with polydactyly (case 25). Coverage plot of chromosome 28 with a 200‐kb window of the affected calf. Note the decrease of the coverage in the end of chromosome 28 (at the level of the blue line). The average coverage across the genome is 14.3 × . Red line: mean genome‐wide average coverage. Blue line: 0.5 × mean coverage

An Angus calf (case 24) was clinicopathological diagnosed with a craniofacial dysmorphism-hydrocephalus-dwarfism syndrome. No single potential candidate variant was identified with the SNVs and small indel prioritization approach in case 24. Accordingly, we examined the presence of larger structural variants and chromosomal abnormalities. Analysis of the depth of coverage along the chromosomes revealed a trisomy of chromosome 23, whereas the parental genomes exhibited normal karyotypes (Fig. 2a). Based on a comparison of the available variant-calling data from the sire and dam, the identified trisomy 23 appears to be due to non-disjunction of homologous chromosomes during the generation of paternal gametes. Heterozygous SNVs along chromosome 28 showed that approximately two-thirds of the variant-containing reads were of paternal origin. Although trisomy 23 has been previously described in calves with dwarfism, the available phenotypical description was limited [108]. Remarkably, human trisomy 6 (corresponding to bovine trisomy 23) [43] is associated with a range of congenital anomalies, including arthrogryposis, syndactyly, facial dysmorphism, ventricular septum defect, intestinal malrotation, and scoliosis [109]. In conclusion, it was determined that this aneuploidy was the most probable cause for this bovine case.

### Polydactyly-Syndactyly-Triphalangism group

A total of five bovine cases were diagnosed with a form of limb malformation belonging to the polydactyly-syndactyly-triphalangism category according to the proposed nosology (trio-approach: cases 25 and 26; single parent-approach: case 27; single cases: case 28 and 29).

#### Polydactyly in cattle

No SNVs and small indel variants were identified in the bovine case 25 diagnosed with polydactyly, assuming either a single recessive or dominant causal variant. Therefore, detection of larger structural variants and chromosomal abnormalities was carried out. Analysis of the depth of coverage along all chromosomes revealed a partial monosomy of chromosome 28 at ~ 2.7 Mb from the chromosome end (Fig. 2b). Both parental genomes had normal karyotypes. Heterozygous SNVs along the hemizygous region showed that approximately two-thirds of the variant-containing reads were of clear paternal origin, indicating that the variant was inherited from the sire. The haploinsufficient region contains 39 annotated genes (See Additional file 3, Table S2) and hemizygosity for these genes may explain the observed phenotype.

Polydactyly in cattle has been frequently reported in the literature, but no clear genetic cause has been found (OMIA:000810-9913). Several SNVs in the *SHH* and *LMBR1* genes have been reported to cause polydactyly in dogs, cats, and chickens (OMIA:000810-9615, OMIA:000810-9685, OMIA:000810-9031). In human distal partial monosomy 10 (corresponding to bovine monosomy 28) [43], clinical findings can vary depending on the location and size of the affected region. However, common phenotypic findings include postnatal growth retardation, developmental delay, microcephaly, and facial dysmorphism [110]. In addition, they can occasionally be associated with syndactyly and brachydactyly [111], although cases of polydactyly have not yet been reported. Therefore, we postulate that this type of monosomy may also cause polydactyly in cattle.

#### Syndactyly in cattle

**Fig. 3:**
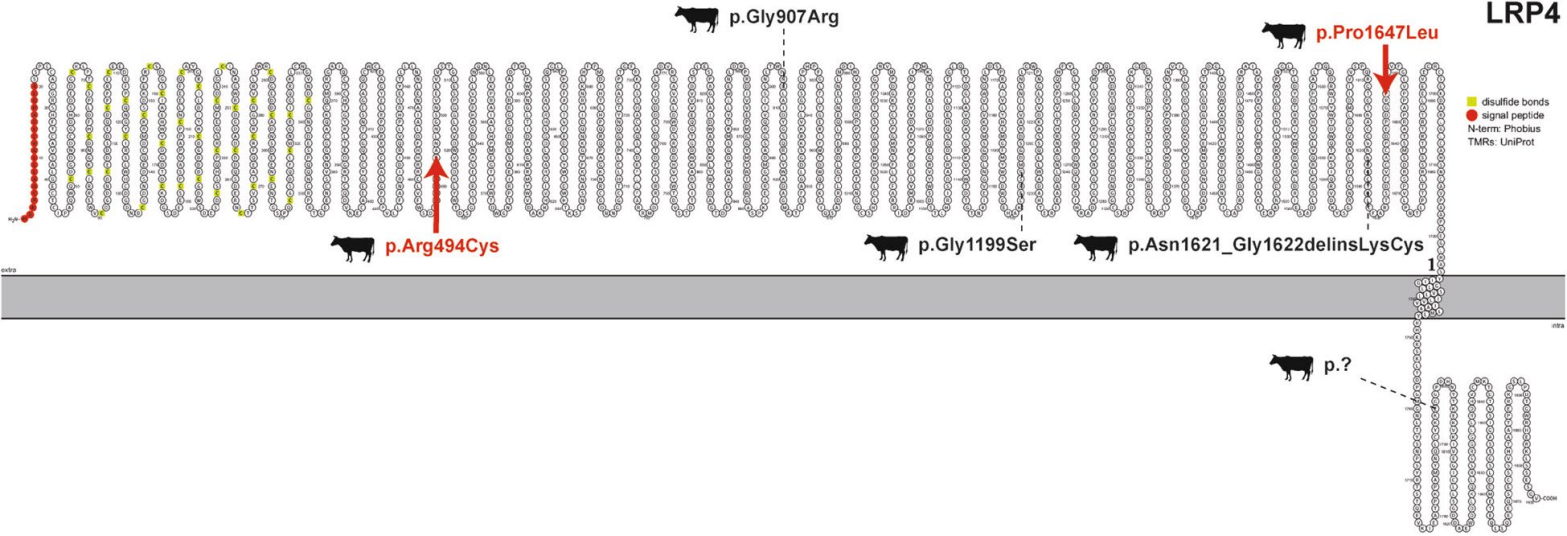
*LRP4*-associated syndactyly in cattle. Schematic representation of the LRP4 protein. The previously reported variants associated with syndactyly are indicated with dotted lines and the newly identified variants in cattle are indicated with red arrows. The novel identified variants include: p.Arg494Cys (case 27) and p.Pro1647Leu (case 26) in a Holstein calves. The previously reported cases include: p.Gly907Arg in Simmental-Charolais calves [113], p.Gly1199Ser in Simmental calves [113], p.Asn1621_Gly1622delinsLysCys in Holstein calves [112] and a splice variant (NM_001077843.1:c.5385 + 1G > A; p.?) in Angus calves [114]

Three Holstein calves (cases 26–28), two females and a male, and one Droughtmaster male (case 29) showed congenital syndactyly of one forelimb. Cases 26 and 27 were paternal half-siblings, whereas case 28 was unrelated for at least four generations. Assuming a recessive MOI, no shared variants were identified that could explain all Holstein cases. However, we did identify the known causal indel variant in exon 33 of *LRP4* (*LRP4* Holstein allele 1: Chr15:g.76800972CdelinsAT; c.4863_4864delinsAT; p.Asn1621_Gly1622delinsLysCys), which has been previously reported in Holstein calves affected by syndactyly [112]. This variant was simultaneously present in a heterozygous state in cases 26 and 27, confirming what was previously reported [113]. In the global cohort, there were five other Holstein individuals carrying a single copy of this variant. In addition, we identified two novel missense variants in *LRP4* in the genomes of case 26 and 27. In case 26, we identified a previously undetected heterozygous missense variant that also affected exon 33 of *LRP4* and alters the encoded amino acid of LRP4 at position 1647, located in the EGF domain (*LRP4* Holstein allele 2: Chr15:g.76800896G > A; c.4940C > T; p.Pro1647Leu) (Fig. 3). A single copy of this variant was present in 403 control genomes (various breeds) but only case 26 had a compound heterozygous *LRP4* genotype carrying both *LRP4* Holstein alleles, 1 and 2. In case 27, another previously undetected heterozygous missense variant was identified in exon 12 of *LRP4* that leads to amino acid exchange of LRP4 at position 494, located in the LY domain (*LRP4* Holstein allele 3: Chr15:g.76819481G > A; c.1480C > T; p.Arg494Cys) (Fig. 3). A single copy of this variant was present in 697 control genomes (various breeds) but only case 27 had a compound heterozygous *LRP4* genotype carrying the *LRP4* alleles 1 and 3. Therefore, these *LRP4* missense variants represent two novel candidate pathogenic alleles for syndactyly in Holstein cattle that were apparently not detected in the PCR-based evaluation of the bovine *LRP4* gene several years ago [113]. Previously, in addition to the causal *LRP4* Holstein allele 1, three other variants affecting the *LRP4* have been associated with syndactyly in Angus, Simmental, and Simmental/Charolais crosses (Fig. 3) [113, 114]. Despite the different variants, the observed phenotype was similar for all cases. Therefore, our findings add to the knowledge of bovine syndactylism by emphasizing the need to consider allelic heterogeneity for *LRP4* variants.

In case 28, assuming a simple recessive MOI, a uncertain significance homozygous missense variant was identified in exon 37 of *KMT2C* that exchanges the encoded amino acid of KMT2C at position 2011 (Chr4:g.114640130G > T; c.6031C > A; p.Pro2011Thr). The syndactyly-affected calf was homozygous and there were only two heterozygous Holstein carriers in the global cohort. *KMT2C* has been associated with Kleefstra syndrome 2 (OMIM 617768), a dominantly inherited neuronal developmental disorder with a wide range of clinical manifestations including short stature, gross motor delay, intellectual disability, central nervous system abnormalities, craniofacial dysmorphisms, and musculoskeletal anomalies including finger and toe malformations [115]. The variants identified in humans with this syndrome were either deletions or nonsense. Therefore, a more severe phenotype could be expected compared to the missense variant identified in the bovine case affected by a milder phenotype. According to Disease Novelty (TIN-X) from Pharos Interface, *KMT2C* is also predicted to be associated with congenital bone disorders including syndactylism, synostosis, and brachydactyly [91].

In case 29, assuming a simple recessive MOI, a homozygous missense variant in exon 2 of *CD4* was identified that exchanges the encoded amino acid of CD4 located in the immunoglobulin subtype 2 domain (Chr5:g.103647362G > A; c.59C > T; p.Pro20Leu). In the global cohort, there were 12 heterozygous variant carriers from five different cattle breeds (Buryat, Charolais, Hanwoo, Mongolian, and Xizang). According to Disease Novelty (TIN-X) from Pharos Interface, *CD4,* like *KMT2C*, is also predicted to be associated with bone development disorders, including syndactylism and brachydactylism [91]. Due to the lack of parents WGS and control genomes from the Droughtmaster breed, the parents were genotyped by PCR. The dam carried the identified homozygous *CD4* variant, while the sire was homozygous wildtype. Parentage control was not possible to perform and therefore it cannot be excluded that the sire is truly the father of the affected calf. These results led us to classify the variant as uncertain significance but most likely not causal. No genome controls were available for the Droughtmaster breed at the time when the WGS analysis was performed. Our results highlight the importance of having parental genomes to exclude variants based on segregation.

Beside *LRP4*-related forms of bovine syndactyly these findings provide further evidence that *KMT2C* may be associated with phenotypically identical non-syndromic forms of bovine syndactylism.

### Dysplasias with multiple joint dislocations

Seven bovine cases (single cases: cases 32–35; single parent-approach: case 36; trio-approach: cases 37 and 38) were diagnosed with various forms of limb dysplasias and craniofacial dysmorphisms, disorders belonging to the dysplasias with multiple joint dislocations category according to the proposed nosology. For two of them (cases 37 and 38), no plausible candidate variant was identified.

#### Craniofacial dysmorphisms and forelimbs skeletal dysplasia in cattle

A stillborn Holstein calf (case 32) was pathologically diagnosed with craniofacial dysmorphisms and forelimbs skeletal dysplasia. Lacking homozygous candidate variants and assuming a dominant MOI, a heterozygous likely pathogenic missense variant was detected in exon 10 of *CCT3,* resulting in an amino acid exchange of CCT3 located in the TCP1 gamma domain (Chr3:g.14497551 T > C; c.907 T > C; p.Tyr303His). It could not be determined whether this variant was due to a spontaneous de novo mutation. In mice, variants in *Cct3* are associated with embryonic and preweaning mortality, and the gene is moderately expressed in the limbs [76, 105]. In addition, *CCT3* is one of the six CCT chaperonin proteins that form a complex with BBS proteins. Variants affecting BBS proteins such as BBS1, BBS2, BBS4, BBS5 and BBS10 have been associated with Bardet-Biedl syndromes reported in humans which are complex and heterogeneous developmental disorders [116]. In Bardet-Biedl syndrome, retinal dystrophy, obesity, polydactyly, genitourinary abnormalities and learning disabilities are common [117]. Given the in silico predictions, expression of the gene, and involvement in the BBsome pathway, we considered that the identified variant in the bovine case could be the cause of the observed disorder.

A Holstein calf (case 33) was clinically diagnosed with craniofacial dysmorphisms, forelimb dislocations, and skeletal dysplasia. Assuming a simple recessive MOI, a homozygous likely pathogenic missense variant in exon 9 of *ITGAE* affecting the von Willebrand factor, type A domain of ITGAE (Chr19:g.24423519A > G; c.986 T > C; p.Ile329Thr) was identified. Using in silico tools, he variant was predicted to significantly affect several molecular mechanisms, including altered stability, transmembrane protein, DNA binding and metal binding, and lead to a loss of allosteric site at I329 [38]. There were no heterozygous animals in the global control cohort. Notably, homozygous *Itgae* mutant mice exhibit decreased body mass, size, and bone mineral content, suggesting that ITGAE may play a role in skeletal disorders [118]. In addition, *ITGAE* physically interacts with six genes (*CDH1*, *EED*, *METTL14*, *RNF123*, *TRIM25*, *TRIM54*) [88]. Remarkably, *CDH1* and *EED* are associated with two dominantly inherited complex skeletal developmental disorders in humans. Patients affected by *CDH1*-related blepharocheilodontic syndrome (OMIM:119580) often show craniofacial dysmorphisms, clinodactyly, and syndactyly [119]. The *EED*-related Cohen-Gibson syndrome (OMIM:617561) is characterized by craniofacial dysmorphisms and several musculoskeletal malformations including joint contractures, flared metaphyses, subluxation of the patella, and *coxa valga* [120]. Given the in silico predictions obtained for the identified variant in *ITGAE,* we speculate that the interaction with the aforementioned genes may be compromised. We report the first bovine variant in *ITGAE* that might be associated with craniofacial dysmorphism, forelimbs dislocation and skeletal dysplasia.

A Chianina heifer (case 34) was clinically diagnosed with craniofacial dysmorphism and forelimbs dysplasia with joint contracture. Assuming a recessive MOI with compound heterozygosity, one heterozygous likely pathogenic missense variant and one intronic heterozygous variant were identified in exon 14 and intron 16 of *CNTNAP1*, respectively (allele 1 in Chr19:g.42747176G > A; c.1495G > A; p.Ala734Thr; and allele 2 in Chr19:g.42748559G > A; c.2531-52G > A). Both variants were not present in the controls. Variants in *CNTNAP1* are known to cause two recessive congenital disorders in humans: lethal congenital contracture syndrome type 7 (LCCS7; OMIM:616286) and congenital hypomyelinating neuropathy type 3 (CHN3; OMIM:618186). Patients affected by these disorders show arthrogryposis multiplex congenital with a non-specific joint involvement, and hypotonia. Other rare non-pathognomonic findings may include craniofacial dysmorphism (e.g. dolichocephaly, brachygnathia, stiff jaw, microcephaly), flexion contractures, and varus deformity of the foot [121]. The LCCS7 has been associated with homozygous frameshift variants, while for CHN3 both homozygous and compound heterozygous missense and nonsense variants have been reported [121, 122]. Interestingly, one of the previously identified variants was an intronic variant upstream of exon 19 of the human *CNTNAP1* that results in a frameshift [122]. Here, we report a Chianina case affected by craniofacial dysmorphism and forelimbs dysplasia with joint contracture that overlaps some clinical features of the human recessive disorders LCCS7 and CHN3. Additional similar Chianina cases could be tested for the identified variants in *CNTNAP1*. Genotyping of hundreds of healthy Chianina controls would help to further validate the identified variants.

#### Forelimbs dysplasia with joint contracture in cattle

Two unrelated Holstein calves (case 35, 36) were clinically diagnosed as forelimb dysplasia with joint contracture. No evidence for a shared recessive causal allele was identified, leading to the hypothesis that de novo mutations may have been the underlying cause of the disorder. In case 35, a heterozygous likely pathogenic disruptive in-frame deletion in *NFE2L1* (Chr19:g.38426902AAGG > A; c.531_533delCCT; p.Leu178del) was detected, while in case 36 we identified a heterozygous likely pathogenic missense variant in exon 12 of *IL16* that exchanges an amino acid located in the PDZ domain of IL16 (Chr21:g.27078178A > G; c.1624A > G; p.Asn542Asp).

The *NFE2L1* gene has been assigned a pLI score of 1, which categories it as a loss-of-function haploinsufficient gene. Therefore, the observed phenotype in case 35 can be attributed to the non-expression of the variant allele. *NFE2L1* plays a regulatory role in osteoclast differentiation, and disruption of this gene can result in reduced bone size and formation. It is predicted to be associated with dysostosis [91, 123, 124]. Based on these findings, we postulate that the identified variant in *NFE2L1* might be the cause of the observed forelimb dysplasia with joint contracture in the Holstein calf.

Remarkably, *IL16* is moderately expressed in the musculoskeletal system and limbs. Among its 112 known interactors, *EFEMP2*, *KDM1A*, *PDLIM7* and *SUV39H1* exhibit direct physical interactions*.* These genes that have been predicted to be associated with bone disorders and disorders with disorganized development of skeletal components [31, 125–127]. In particular, *EFEMP2* is associated in humans with a syndromic condition known as *cutis laxa* type 1B (OMIM:614437) that is characterized by inelastic skin (*cutis laxa*), arterial tortuosity, aneurysms and stenosis, joint laxity, contractures of fingers, and arachnodactyly [128]. In addition to *cutis laxa*, *Efemp2* mutant mice also show abnormal tendon collagen fibrils and forelimb morphology [129, 130]. Considering these findings, we postulate that forelimb dysplasia with joint contracture in the Chianina heifer may be attributed to an impaired interaction between *IL16* and the aforementioned interactors. Further functional studies would be needed to validate this hypothesis.

### Skeletal congenital disorders with unknown genetic cause

In eight bovine cases diagnosed with 6 disorders, no candidate variant could be identified. Cases 16, 20 and 23 were diagnosed with primordial proportionate dwarfism, primordial disproportionate dwarfism and arachnomelia, respectively, and were included in the Primordial dwarfism and slender bones group. Cases 30 and 31 showed congenital rickets and were thus grouped with the disorders of bone mineralization category. Cases 37 and 38 were clinically diagnosed with forelimb dysplasia with joint contracture and were included in the dysplasias with multiple joint dislocations group. Ultimately, case 39 was diagnosed with hemifacial microsomia with hemivertebrae and was thus grouped in the vertebral and costal dysostoses category. Further clinicopathological and genomic details of these cases can be found, respectively in Additional file 1, Figures S1 to S20 and Additional file 2, Table S1.

The absence of a potential genetic diagnosis in these cases can be due to a number of factors, including methodological limitations such as the incomplete annotation of the bovine genome, the emphasis on coding variants in selected candidate genes based on literature searches, the non-survey for intermediate sized structural variants, challenges associated with the used short-read WGS technology, inaccuracies in read alignment and variant calling [131], or the potential influence of epigenetics or non-genetic factors. This may include intra-uterine infections with teratogenic agents such as bovine viral diarrhea virus [132], blue tongue virus and related orbiviruses [133], *Orthobunyaviruses* of the Simbu serogroup (e.g. Schmallenberg virus, Akabane virus, Peaton virus) [134] and Toxoplasma gondii [135]. Moreover, non-genetic factors that might be associated with congenital skeletal disorders include mineral (e.g. manganese) [136] and oligoelements (e.g. selenium) [137] deficiency or ingestion of teratogenic plants (e.g. lupine) during the gestational period [138].

## Conclusions

This study reviews a series of bovine and ovine cases affected by various skeletal disorders. The WGS-based veterinary precision medicine approach revealed considerable allelic and genetic heterogeneity of the described phenotypes in terms of mode of inheritance as well as the variant type. We propose, for the first time, a consensus categorization of genetic skeletal disorders in cattle and sheep, which may be useful for more accurate differential clinicopathological diagnosis. We highlight how WGS proposes its implementation in genetic disorder surveillance, allowing the differentiation between sporadic genetic defects and recessive disorders of importance to population health. The candidate likely causal alleles for recessive disorders should be considered in breeding programs to exclude risk matings.

Our findings contribute to a more comprehensive understanding of the potential associations between inherited skeletal disorders and known or novel candidate genes. As a result, their study offers significant potential for the rapid discovery of new spontaneous large animal models for human disease. Moreover, we highlight the impact of the discovery of genes that cause rare disorders, from clinical and pathological diagnosis to the insights achieved into biological mechanisms and common disorders.

## Supplementary Information

Additional file 1: Figure S1. Achondrogenesis type II in a Holstein calf. (A) Mild form of achondrogenesis type II in case 1: note the short compact body and muzzle, the bilateral valgism of both fore- and hindlimbs, and the slight protrusion of the tongue. (B) 3D reconstruction of skull CT scan shows a deformation of the cranial vault which appear bulging. (C) and (D) Sagittally reconstructed CT image (C) and 3D reconstruction CT scan (D) of the one forelimb reveal a marked reduction in the length of all bones, with a stubby profile and widened metaphysis. (E) Sagittally reconstructed CT image of the one hindlimb shows the same abnormalities described in the forelimbs. (F) 3D reconstruction CT scan of the pelvis and hindlimbs reveals a pronounced valgism. Case 1. Figure S2. Achondrogenesis type II in sheep. (A) Achondrogenesis type II-affected lamb: note the short compact body and limbs and the malformed head. (B) Radiographic image of a hind limb illustrating the severe abnormalities of most bones. The femur (F), tibia (T) and metatarsal bones (MT) were only identifiable by their location, as only their irregularly shaped diaphysis are seen. The phalangeal bones were well developed and of normal shape. (C) Radiographic image of a skull illustrating dysplasia of the viscerocranium with shortening of the maxillary bones and protrusion of the tongue. Case 2. Figure S3. Osteogenesis imperfecta in a Holstein calf. (A) The calf is of reduced size. The long bones are abnormally thin with bilateral hyperextension of the tarsal joints. There is abnormal angulation of the proximal part of the right metatarsus due to a closed transverse fracture. (B) Radiograph of the right hindlimb showing an acute non-articular comminuted fracture of the proximal diaphysis of tibia and fibula and an acute transverse fracture of the proximal diaphysis of the metatarsal bones. The cortical bone is abnormally thin. (C) Radiograph of the left thorax showing multiple congenital rib fractures. Some fractures have smooth modeled callus formation being consistent with advanced stages of fracture healing, while others have little callus formation and sharp margins suggesting recent fractures. All ribs appear thickened. The mid and distal parts of the ribs are greatly expanded and have a no visible cortex. Some ribs have a radiating trabecular bone pattern, multiple horizontal radiopaque parallel lines, and amorphous mineralization. Other ribs have a more organized normal appearing bone structure but without a discrete cortex. Case 8. Figure S4. Osteogenesis imperfecta in Stabilizer calves. (A) Note the domed skull, and distorted or twisted limbs. (B) Note the congenital fracture in the right forelimb. Case 9. Figure S5. Osteopetrosis and brachygnathia inferior in a crossbred calf. (A) Affected calf showing inferior brachygnathism, protrusion of the tongue, slight thoracolumbar scoliosis and twisted limbs. (B) Note the distal exposed fracture in the left metacarpal bone. (C) Note the radio-ulna fracture and the angular deformities in the radio-ulna and the thinness of the distal part of the humerus body (proximally to the condyles). In addition, note the enlarged articular spaces between the carpal bones. (D) Note the metatarsal fracture. Case 14. Figure S6. Caudal and thoracic vertebral and viscerocranial malformations in a Holstein heifer. (A) and (B) Note the crooked tail deviated to the left side and a narrow viscerocranium. Case 15. Figure S7. Primordial proportionate dwarfism in a Simmental calf. (A) Comparison between a 3-month-old proportionate dwarf Simmental calf (left) to a normal 2-week-old Simmental calf (right). Note the almost equal size. (B) Proportionate dwarf at age of 3 months. Case 16. Figure S8. Primordial proportionate dwarfism in a Simmental heifer. Case 17. Figure S9. Primordial disproportionate dwarfism in Holstein cattle. Note disproportionate short stature with limb shortening case 20 in (A) and case 21 in (B). Figure S10. Figure S10. Primordial proportionate dwarfism in a crossbred calf. Note the difference between the proportional dwarf calf at four months of age (first plan) compared to healthy one-month-old calves (second plan). Figure S11. Arachnomelia in a Holstein calf. (A) Note the dolichostenomelia and angular deformities of all limbs. (B) Note the more pronounced angular deformities in the distal forelimbs and marked bilateral hyperextension of the fetlocks. Case 23. Figure S12. Craniofacial dysmorphism-hydrocephalus-dwarfism syndrome in an Angus calf. (A) Note the superior brachygnathism, the dome shaped neurocranium, exophthalmos and shortening and widening of the limbs. (B) Particular of the head. Note superior brachygnathism, the dome shaped neurocranium, and exophthalmos. Case 24. Figure S13. Polydactylism in a crossbred calf. (A) Note the abaxial wall of the medial digit of the left front foot curled distal and plantar under the foot with a small, extra digit next to the medial digit and the left forelimb shows a duplication at the carpal level. (B) Radiograph of the right forelimb showing a duplication of the limb at the carpal level. Note the presence of two metacarpi. Figure S14. Syndactyly in a Droughtmaster calf. (A) and (B) Note the syndactyly of the right forelimb and the partial syndactyly in the left forelimb. Boths back feet are normal. Case 29. Figure S15. Craniofacial dysmorphisms and forelimb skeletal dysplasia in a Holstein calf. (A) Note that both forelimbs are dysplastic with bilateral almost symmetric arthrogryposis and medial rotation if the carpal joint. The two main digits and the medial dewclaw were developed in the left forelimb, while only the medial digit and the corresponding dewclaw were present in the right forelimb. (B) Radiograph of the right forelimb showing absence of the radius. Additionally, luxation of the shoulder joint a poorly developed ulnar notch, there is minimal contact between the ulna and humeral condyle, ulna’s mid diaphysis is thickened and in its distal part it is expanded to form a broad based articulation with the carpus, metacarpal III is present and articulates distally with normal appearing phalanges and a short thin bone structure (0.3 cm diameter by 1.75 cm length) is present adjacent to proximal metacarpal III; this is presumed to be a rudimentary metacarpal IV. Case 32. Figure S16. Craniofacial dysmorphisms, forelimbs dislocations and skeletal dysplasia in a Holstein calf. (A) and (B) Note axial limb malalignment and the lateral rotation of the radial bones of both forelimbs. (C) Dorsoplantar radiography of the right hindlimb. Note the prominent endorotation of the tibia starting proximal at the tibial apophysis with medial curving of the tibial diaphysis. Axial rotation results in a malalignment of the femoro-tibial and tarso-metatarsal joints. Case 33. Figure S17. Craniofacial dysmorphism and forelimbs dysplasia with joint contracture in a Chianina heifer. (A) Note the craniofacial dysmorphism, protrusion of the tongue and the bilateral flexural deformities of the forelimbs characterized by lateral rotation and bowing with join contracture. (B) Particular of the head. Note the craniofacial dysmorphism including shortening of the viscerocranium, concavity of the nasal bone, brachygnathia superior. Case 34. Figure S18. Forelimbs dysplasia with joint contracture in a Chianina heifer. (A) and (B) Note the bilateral elbow abduction and flexor deformity on the carpus bilaterally. Figure S19. Forelimbs dysplasia with joint contracture in Limousin calves. Note the carpus valgus. Case 38. Figure S20. Microsomia with hemivertebrae in a Rendena calf. (A) Note the unilateral microtia (left external auricle), divergent strabismus in the left eye and facial asymmetry with deviation of the median plane to the left. Note that the ears are pendulous, pointing downwards more markedly on the left. Note the atrophy of the masseter and flaccidity of the left lip (drooping of the mouth). (B) Note the short neck and reduced nutritional status.

Additional file 2: Table S1. Cases phenotypical information, congenital skeletal disorders classification, associated genes, mode of inheritance and variants characterization.

Additional file 3: Table S2. Annotated genes encompassed in the chromosome 28 partial monosomy in the crossbred calf with polydactyly (case 25).

## Data Availability

All WGS data is available on the European Nucleotide archive under the Swiss Comparative Bovine and Ovine Resequencing Projects (accession numbers PRJEB30931, PRJEB83441 and PRJEB18113). The individual sample accession numbers of all cases used in the current study are reported in Additional file 2, Table S1.
