# Supplementary material for: Exploring skeletal disorders in cattle and sheep: a WGS-based framework for diagnosis and classification
Source: Genet Sel Evol. 2025 Sep 25;57:51. doi: 10.1186/s12711-025-01002-z (PMC12465177; doi:10.1186/s12711-025-01002-z)
Supplement: Supplementary file 1 — Additional file 1: Figure S1. Achondrogenesis type II in a Holstein calf. (A) Mild form of achondrogenesis type II in case 1: note the short compact body and muzzle, the bilateral valgism of both fore- and hindlimbs, and the slight protrusion of the tongue. (B) 3D reconstruction of skull CT scan shows a deformation of the cranial vault which appear bulging. (C) and (D) Sagittally reconstructed CT image (C) and 3D reconstruction CT scan (D) of the one forelimb reveal a marked reduction in the length of all bones, with a stubby profile and widened metaphysis. (E) Sagittally reconstructed CT image of the one hindlimb shows the same abnormalities described in the forelimbs. (F) 3D reconstruction CT scan of the pelvis and hindlimbs reveals a pronounced valgism. Case 1. Figure S2. Achondrogenesis type II in sheep. (A) Achondrogenesis type II-affected lamb: note the short compact body and limbs and the malformed head. (B) Radiographic image of a hind limb illustrating the severe abnormalities of most bones. The femur (F), tibia (T) and metatarsal bones (MT) were only identifiable by their location, as only their irregularly shaped diaphysis are seen. The phalangeal bones were well developed and of normal shape. (C) Radiographic image of a skull illustrating dysplasia of the viscerocranium with shortening of the maxillary bones and protrusion of the tongue. Case 2. Figure S3. Osteogenesis imperfecta in a Holstein calf. (A) The calf is of reduced size. The long bones are abnormally thin with bilateral hyperextension of the tarsal joints. There is abnormal angulation of the proximal part of the right metatarsus due to a closed transverse fracture. (B) Radiograph of the right hindlimb showing an acute non-articular comminuted fracture of the proximal diaphysis of tibia and fibula and an acute transverse fracture of the proximal diaphysis of the metatarsal bones. The cortical bone is abnormally thin. (C) Radiograph of the left thorax showing multiple congenital rib fractures. S [file 12711_2025_1002_MOESM1_ESM.pdf]

Additional File 1. Clinicopathological description of the included bovine and ovine cases affected by congenital skeletal disorders.

Type 2 collagen disorders

Achondrogenesis type II in a Holstein calf

A 5-days-old female Holstein calf (case 1) with a body weight (BW) of 18 kg was referred to the Clinic for Ruminants of the University of Bologna, Italy due to short stature and malformed limbs. The calf was the result of an artificial insemination of a purebred Holstein sire on a Holstein dam. The calf was delivered at gestation day 276 (normal average gestation length for Holsteins is approximately 281 days). The parents were not related within at least four generations. On the clinical examination, morphologically, the calf showed generalized disproportionate dwarfism resembling bovine achondrogenesis type II. (Supplementary Figure S1, A). The neck and body appeared shortened and compact. The limbs showed bilateral symmetric shortening, which especially affected the bones proximal to the phalanges, giving the limbs a compact appearance. The fetlock joints were stiff and rotated medially and were persistently brought into flexion at an angle of approximately 30°. Valgism of both fore- and hindlimbs was observed bilaterally. The carpal and tarsal joints were stiff and held flexed. The head was disproportionately large compared to the body and the viscerocranium was shortened. The calf showed slight protrusion of the tongue. Abnormal cranial nerve reflexes were not noticed. The calf showed tachypnea (50 rpm), tachycardia (150 bpm) and normothermia (38.4°C). No abnormalities were noticed at thoracic auscultation except for increase cardiac and respiratory rates. The calf also showed recurrent abdominal blotting and diarrhea.

Radiological examination revealed normally structured phalangeal bones, malformed femoral greater trochanter, a rudimentary patella, non-fused metacarpal and metatarsal bones II and IV with a slight abaxial rotation. The CT scan confirmed the findings observed in the radiographic examination of the appendicular skeleton. In particular the long bones of both forelimbs and hindlimbs appeared shorted associate with a metaphyseal flaring giving a "trumpet bone" appearance. Additionally, the cranial CT revealed an altered frontonasal profile with a rather curved morphology of the frontal and occipital bones.

Both clinical and imaging finding were consistent with a form of achondrogenesis. Due to the poor prognosis the calf was euthanized at age of one month but was not necropsied. Genetic analysis confirmed that the calf had achondrogenesis type II.

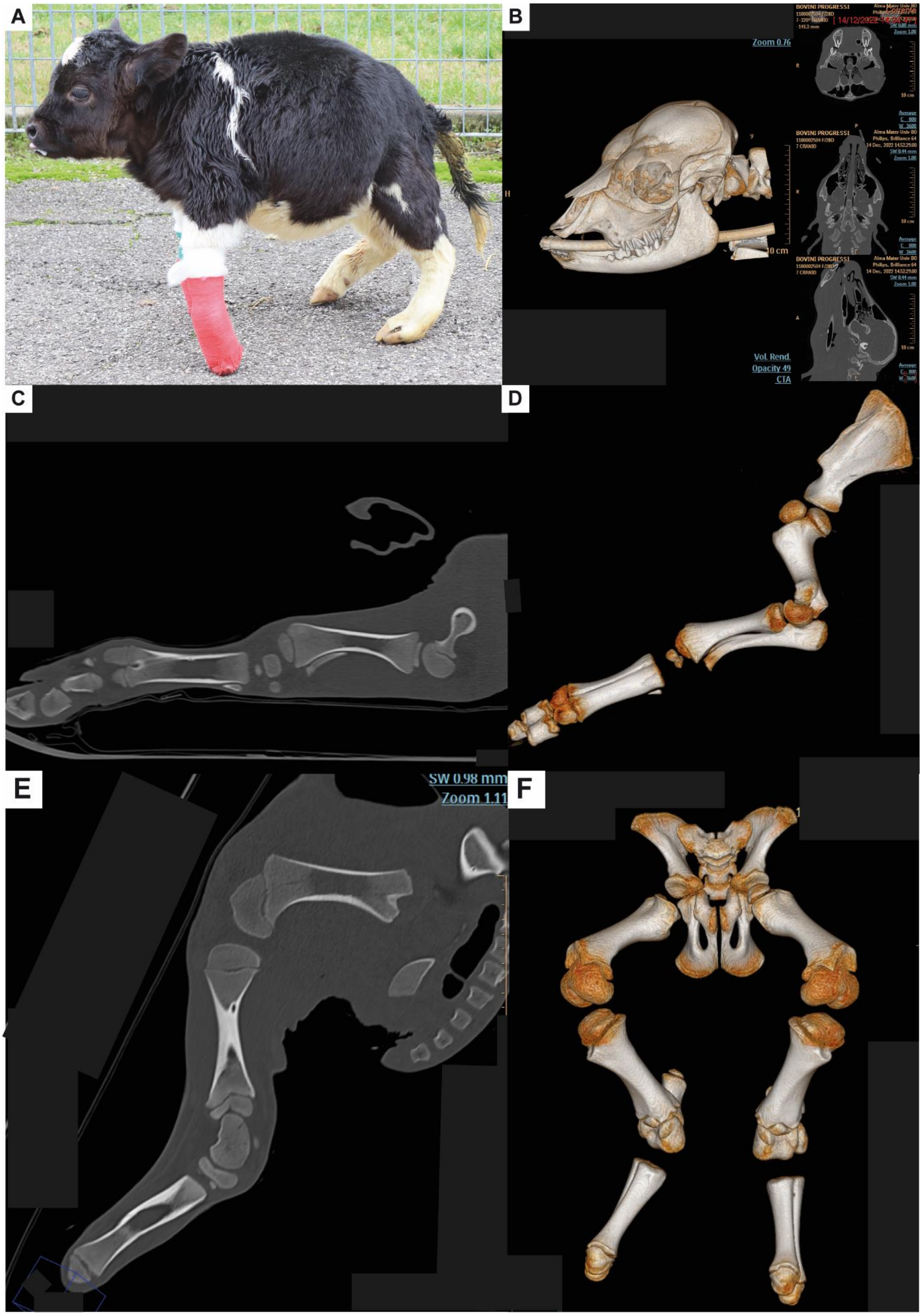

Supplementary Figure S1. Achondrogenesis type II in a Holstein calf. **(A)** Mild form of achondrogenesis type II in case 1: note the short compact body and muzzle, the bilateral valgism

of both fore- and hindlimbs, and the slight protrusion of the tongue. **(B)** 3D reconstruction of skull CT scan shows a deformation of the cranial vault which appear bulging. **(C)** and **(D)** Sagittally reconstructed CT image (C) and 3D reconstruction CT scan (D) of the one forelimb reveal a marked reduction in the length of all bones, with a stubby profile and widened metaphysis. **(E)** Sagittally reconstructed CT image of the one hindlimb shows the same abnormalities described in the forelimbs. **(F)** 3D reconstruction CT scan of the pelvis and hindlimbs reveals a pronounced valgism. Case 1.

Achondrogenesis, type II in crossbred sheep

Six paternal half-sibling crossbred lambs (cases 2-7) from a single English flock were investigated at Sheep & Beef Health Services, United Kingdom and the Animal and Plant Health Agency (APHA), UK due to abortion and short stature. All lambs were progeny of the same Charollais ram. The flock had 480 commercial ewes and replacement females are mostly purchased and originate from multiple flocks. Gross-pathology and histopathology were performed. Morphologically, all cases showed generalized disproportionate dwarfism resembling bovine achondrogenesis type II. The body of the affected lambs appeared shortened and compact. The limbs showed bilateral symmetric shortening, which especially affected the bones proximal to the phalanges, giving the limbs a compact appearance (Supplementary Figure S2, A). The head of all cases had dysplasia of the viscerocranium with shortening of the maxillary bones and protrusion of the tongue. Radiographic examination of a hindlimbs revealed severe bone abnormalities of most bones. Of the femur, tibia and metatarsal bones only their irregularly shaped diaphysis was seen, and these bones were only identifiable by their location (Supplementary Figure S2, B). The phalangeal bones were well developed and of normal shape. The morphological craniofacial features observed in the macroscopical examination were further confirmed by the radiological examination (Supplementary Figure S2, C). Histologically, failure of endochondral ossification leading to a chondrodystrophy was observed. In the femur there was absence of ossification centres in the epiphysis. There was mild disorganization of chondrocyte cords within the proliferative and hypertrophic areas of the physis along with the formation of nodular hypertrophic chondrocyte isles. There was also complete absence of the primary trabecular latus, foci of immature bone formation, and enlargement of the secondary trabeculae. In the epiphyseal cartilage of the distal femur additionally abnormal collagen fibre deposition within the extracellular matrix was observed. Bone marrow was unremarkable. In the tibia vascular channels were broad and tortuous throughout the epiphyseal cartilage. Moderate disorganization of chondrocyte cords within the

proliferative and hypertrophic areas of the physis was present along with the formation of nodular hypertrophic chondrocyte isles which occasionally were also visible within the primary trabecular latus. This latus was severely narrowed/ absent and presents foci of immature bone formation. Secondary trabeculae were moderate to severely thickened, and fibrous tissue formation amongst trabeculae (myelofibrosis) was seen. In the spinal cord, a latero-lateral compression of the C1 section with an enlarged central canal, tentatively lined by a multifocally attenuated ependyma was observed. Both gross and histopathological finding were consistent with a form of achondrogenesis.

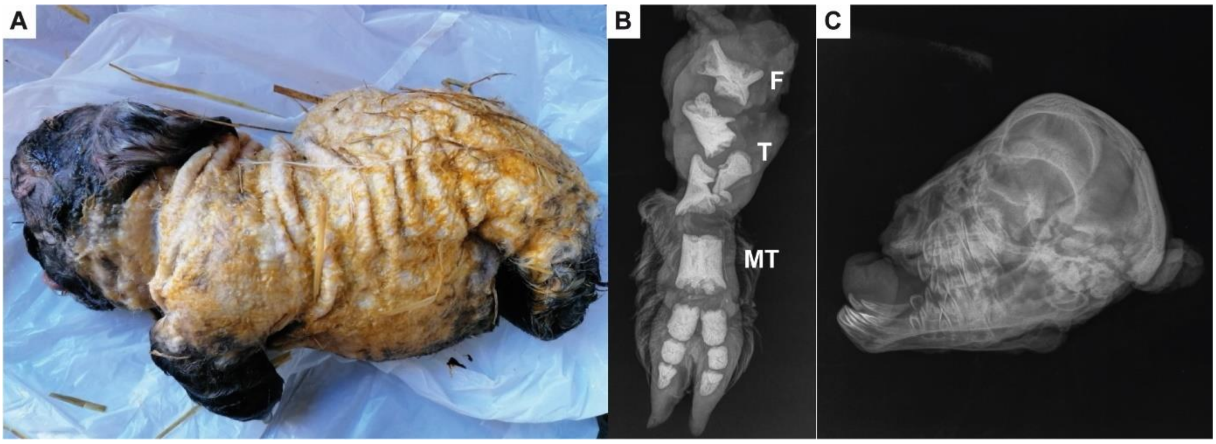

Supplementary Figure S2. Achondrogenesis type II in sheep. **(A)** Achondrogenesis type II-affected lamb: note the short compact body and limbs and the malformed head. **(B)** Radiographic image of a hind limb illustrating the severe abnormalities of most bones. The femur (F), tibia (T) and metatarsal bones (MT) were only identifiable by their location, as only their irregularly shaped diaphysis are seen. The phalangeal bones were well developed and of normal shape. **(C)** Radiographic image of a skull illustrating dysplasia of the viscerocranium with shortening of the maxillary bones and protrusion of the tongue. Case 2.

Osteogenesis imperfecta and bone fragility group

Osteogenesis imperfecta in a Holstein calf

A stillborn female Holstein calf (case 8) was submitted to the University of Copenhagen, Denmark for postmortem examination as part of the Danish surveillance program for bovine genetic diseases as fractures were evident upon delivery. The calf was born at gestation day 269 as the results of an artificial insemination of a purebred Holstein sire on a Holstein dam. The parents were related within two generations. The carcass had been frozen before submission and was examined after thawing.

The calf had reduced size with a body weight of 26.3 kg but was of normal proportions and body condition (Supplementary Figure S3. A). The long bones appeared abnormally thin and had multiple transverse fractures . The stifle and tarsal joints showed bilateral hyperextension. The carpal joint had bilateral increased mobility due to increased laxity of tendons and ligaments. Multiple rib fractures were present and of various age ranging from acute fractures to chronic fractures with excessive osseous callus formation. The teeth and sclera had a slight blue color.

For radiology, specimens consisting of the left and right thoracic wall and the fore- and hindlimbs were selected. Images of the hindlimbs also showed multiple fractures. Each tibia had a comminuted, non-articular diaphyseal fracture with sharp margins and no callus formation. Fractures were also found in the metatarsal region of both limbs and involved the fused metatarsal III and IV. Again, these fractures had sharp edges and no callus formation. Images of the thoracic limb showed an incomplete hairline fracture of the distal diaphysis of one of the metacarpal bones. The margins of this fracture were sharp and without callus formation (Supplementary Figure S3. B). Radiographs of the ribs showed multiple fractures of which some had smooth modeled callus being consistent with advanced stages of fracture healing, others had none or limited callus formation and sharp margins suggesting recent fractures (Supplementary Figure S3. C). All ribs appeared thickened and the mid and distal parts were greatly expanded and with no visible cortex. Some ribs had a radiating trabecular bone pattern, multiple horizontal radiopaque parallel lines, and amorphous mineralization, while other ribs had a more organized normal appearing bone structure but without a discrete cortex.

Histopathological examination of sagittal sections of the primary developing first incisor with surrounding alveolar bone was performed on the calf and an age-matched healthy control. This revealed a significant reduction in dentin thickness compared to the control. The predentin layer exhibited abnormal characteristics, including increased thickness, granulation, and a wavy interface with the dentin. The dentinal tubules were notably sparse, with increased diameter and irregular course through the dentin. Cellular inclusions were occasionally observed within the dentin matrix. Odontoblasts demonstrated marked morphological alterations, including reduced size and loss of their characteristic columnar morphology. Similarly, the bone tissue showed affected osteoblasts with reduced cell size and less prominent Golgi apparatus (negative Golgi image). The osteocyte lacunae were characterized by increased size, irregular morphology, and higher frequency throughout the bone matrix.

Gross, radiological and histopathological findings were consistent with the characteristic features of osteogenesis imperfecta and dentinogenesis imperfecta.

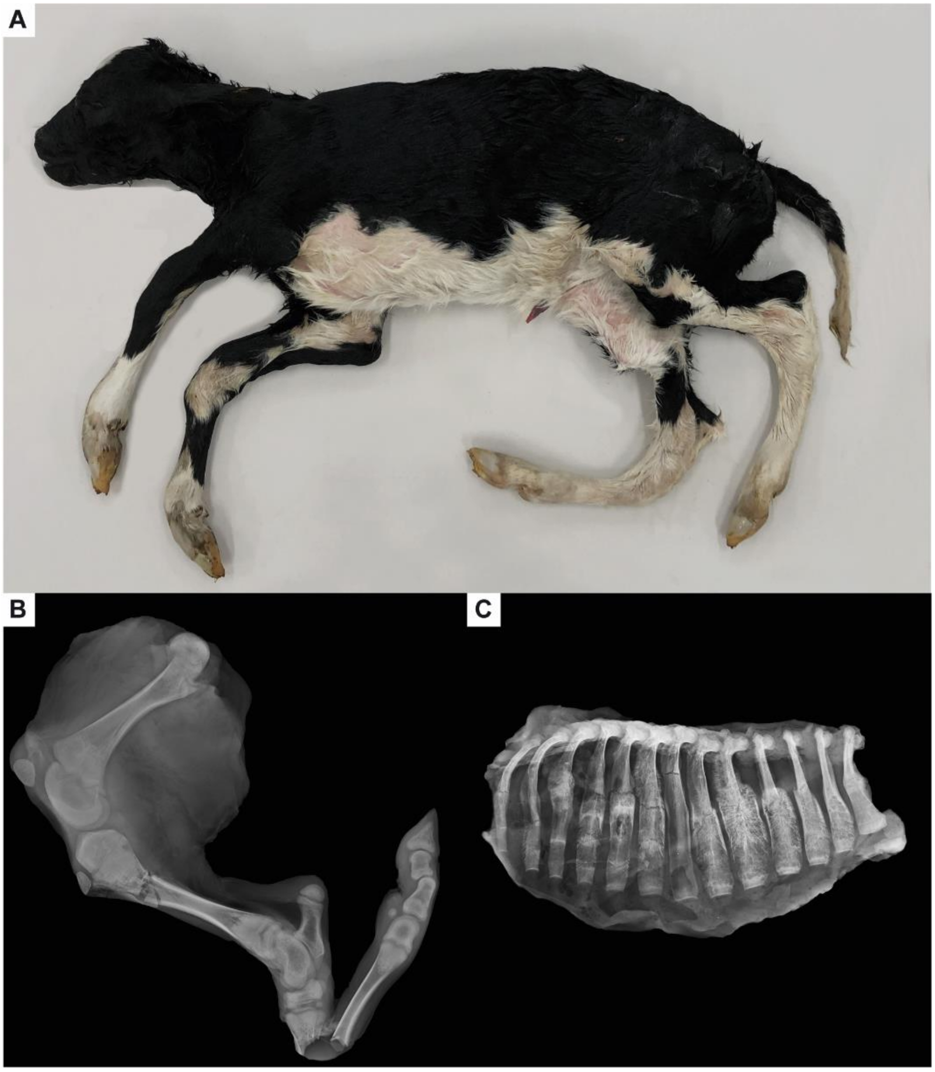

Supplementary Figure S3. Osteogenesis imperfecta in a Holstein calf. **(A)** The calf is of reduced size. The long bones are abnormally thin with bilateral hyperextension of the tarsal joints. There is abnormal angulation of the proximal part of the right metatarsus due to a closed transverse fracture. **(B)** Radiograph of the right hindlimb showing an acute non-articular comminuted fracture of the proximal diaphysis of tibia and fibula and an acute transverse fracture of the proximal diaphysis of the metatarsal bones. The cortical bone is abnormally thin. **(C)** Radiograph of the left thorax showing multiple congenital rib fractures. Some fractures have smooth modeled callus formation being consistent with advanced stages of fracture healing, while others have little callus formation and sharp margins suggesting recent fractures. All ribs appear thickened. The mid and distal parts of the ribs are greatly expanded and have a no visible cortex. Some ribs have a radiating trabecular bone pattern, multiple horizontal

radiopaque parallel lines, and amorphous mineralization. Other ribs have a more organized normal appearing bone structure but without a discrete cortex. Case 8.

Osteogenesis imperfecta in Stabilizer calves

Two stillborn paternal half-siblings Stabiliser (a composite breed) calves (male and female, Cases 9 and 10 respectively) from two different English herds were submitted to the APHA due to congenital fractures and stillbirth. The calves were progeny of the same sire. The calves showed similar gross pathology findings which included a domed skull, long bone limb and rib fractures, and distorted or twisted limbs (Supplementary Figure S4, A and B). There was also shortened mandible (inferior brachygnathism) and an open fontanelle in the caudo-occipital region. Another consistent feature was a blue/grey color of the sclera.

Histopathological examination of bones from each of the calves identified normal progression of endochondrial ossification at the physes, but the cortices were thin and porous with only mild osteoid deposition and remodeling of the spongiosa, and notably thin and poorly connected primary trabeculae extending deep into the metaphysis. The rib fractures had spicules of woven and laminar bone, arranged haphazardly, with proliferating fibroblasts, among a loose myxoid matrix, and multifocal hemorrhages. The skull had interconnected, radiating spicules of lamella and woven bone. In general, the epiphyseal trabeculae were reduced in size and number and the cortical bone appeared thinner. These histopathological features were consistent with osteogenesis imperfecta.

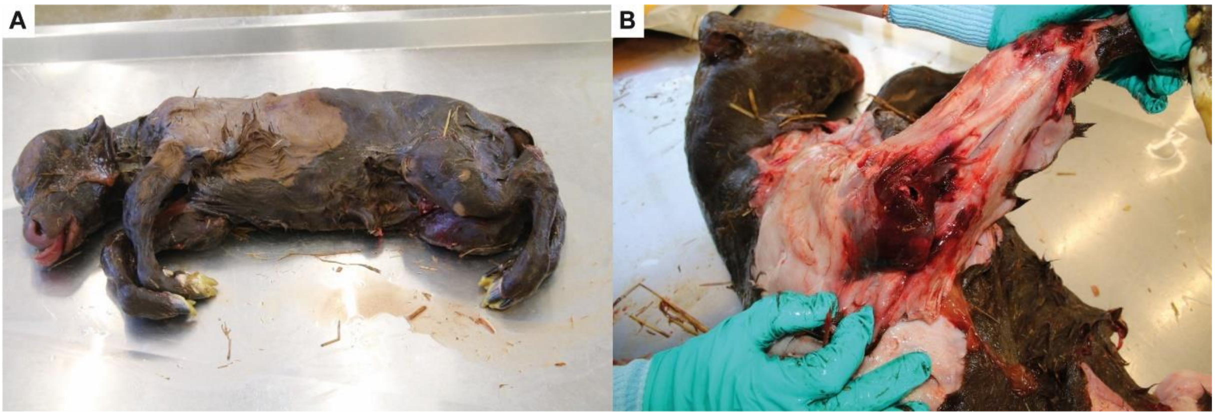

Supplementary Figure S4. Osteogenesis imperfecta in Stabilizer calves. **(A)** Note the domed skull, and distorted or twisted limbs. **(B)** Note the congenital fracture in the right forelimb. Case 9.

Osteogenesis imperfecta in crossbred sheep

Two one-month-old, female, paternal half-sibling, crossbred lambs (Cases 11 and 12 respectively) from the same English lowland flock were submitted to the APHA for post-mortem examination to investigate a problem of hindlimb weakness / paralysis, affecting a total of 14 lambs from a group of 34. All lambs had been sired (natural mating) by a newly purchased Charollais ram and were born to Lleyn x Romney ewes. Clinical signs were first noticed when the lambs had reached 2-3-weeks of age.

Gross findings in both lambs were similar and included multiple, mid-shaft fractures of the ribs, with evidence of localized callous formation and mid-shaft fracture of a single femur with frank hemorrhage into the surrounding musculature and soft tissues. In addition, in both cases the bones of the skeleton were notably soft and pliable. Nutritional bone disease including copper deficiency, lead toxicity and rickets were ruled out on biochemical testing of liver, kidney and serum respectively and there was no evidence of concurrent enteric disorders which may have impacted bone growth and density.

Histopathological examination of the long bones and ribs from each lamb identified normal cartilage and endochondial ossification at the physes but the cortices of the long bones were thin and porous, and the trabeculae were thin and poorly connected. Subjectively, there was reduced deposition of osteoid along newly formed bone – which was evident in the primary and secondary spongiosa of intact bone and at fracture sites – and the trabeculae were lined by hypertrophied osteoblasts.

EDTA blood samples were subsequently collected from an additional affected alive lamb that was of notably smaller stature, fine boned and had an elongated head shape (Case 13).

Osteopetrosis and related osteoclast disorders

Osteopetrosis and brachygnathia inferior in a crossbred calf

A crossbred female calf (case 14) with a BW of 28 kg was delivered at gestation day 270. The pregnancy was the result of the insemination of a purebred Angus sire on a Holstein dam. The calf died spontaneously 30 minutes after birth. The calf was then submitted to the Institute of Genetics, Vetsuisse Faculty, University of Bern, Switzerland due to the presence of skeletal malformations. Brain samples were tested for bovine viral diarrhea virus (BVDV), Schmallenberg virus (SBV) and bluetongue virus (BTV) using PCR. Tests were negative.

Gross pathology revealed inferior brachygnathism, protrusion of the tongue, slight thoracolumbar scoliosis and twisted limbs (Supplementary Figure S5, A). The viscerocranium

was slightly narrowed. The spinal column showed scoliosis in the thoracolumbar region. Acute rib fractures and distal fractures in the metacarpal and metatarsal bones and in the radio-ulna were noticed bilaterally (Supplementary Figure S5, B, C and D). The radio-ulna bones were abnormally curved and in the distal part of the humerus body (proximally to the condyles) the bone was very slim (Supplementary Figure S5, C). The articular spaces between the carpal bones were enlarged bilaterally contributing to the increase of the joint mobility. The metatarsal bones were curved, and the metatarso-phalangeal joints showed hyperextension with reduced mobility on flexion but abduction- and adduction-movements were increased. The hock joints showed also increased mobility with increased adduction- and abduction-movements. The angle between tarsus and metatarsus was increased bilaterally (around 90º). Ectopia of the spiral loop of the ascending colon with grade III was noticed. Stenosis of the truncus pulmonalis and the ductus arteriosus were observed. At the midline section of the head, the cerebellum was noticed to be herniated into the vertebral channel (Chiari-malformation type I).

Histologically, the medullary cavity of the examined bones was nearly completely (rib) to partly (metacarpus, medullary cavity nearby the metaphysis) filled by numerous, rather thin, irregularly shaped, partly anastomosing trabeculae composed of woven bone and cartilage tissue. These trabeculae were covered by rather scant osteoblasts (less numerous in the metacarpus sample). Osteoclasts were in both samples rare to absent. The histological features of the bones were compatible with osteopetrosis.

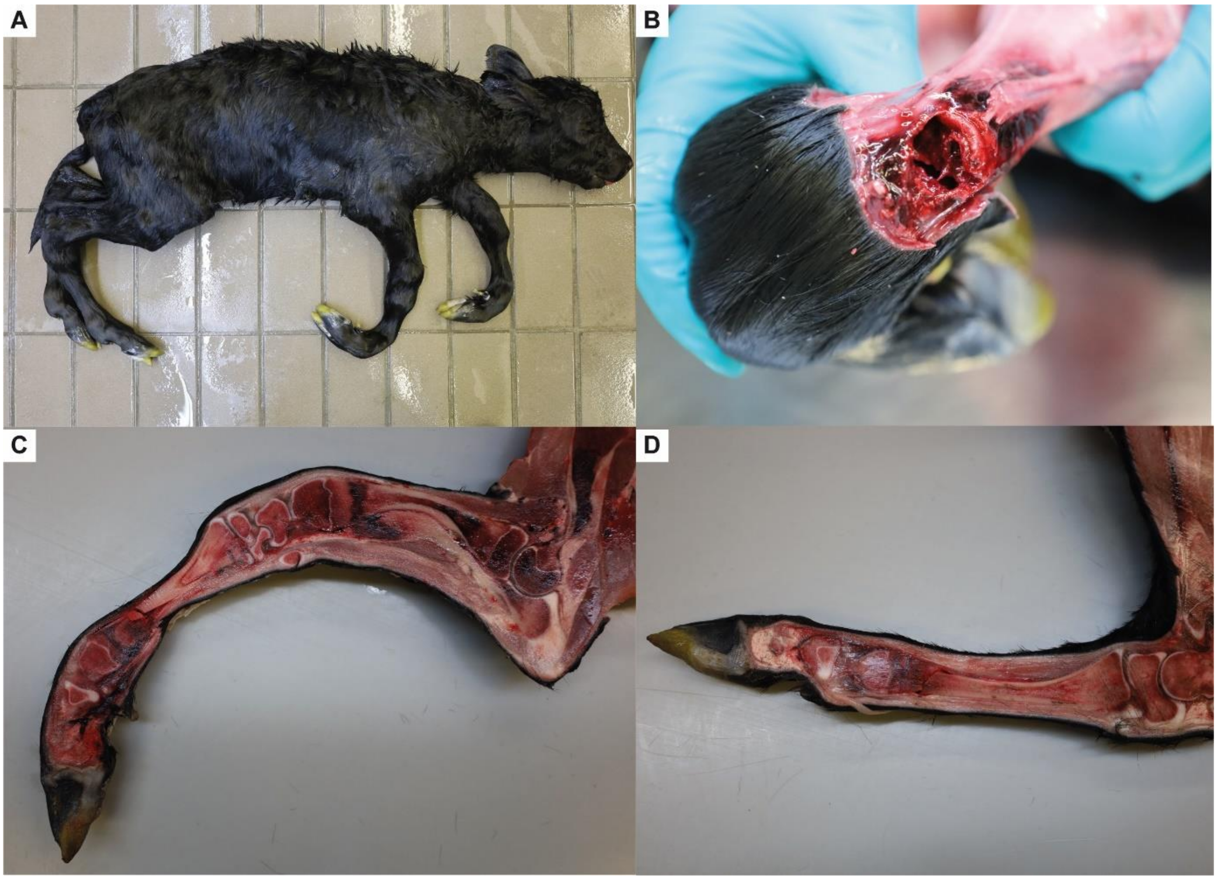

Supplementary Figure S5. Osteopetrosis and brachygnathia inferior in a crossbred calf. **(A)** Affected calf showing inferior brachygnathism, protrusion of the tongue, slight thoracolumbar scoliosis and twisted limbs. **(B)** Note the distal exposed fracture in the left metacarpal bone. **(C)** Note the radio-ulna fracture and the angular deformities in the radio-ulna and the thinness of the distal part of the humerus body (proximally to the condyles). In addition, note the enlarged articular spaces between the carpal bones. **(D)** Note the metatarsal fracture. Case 14.

Sulfation disorders

Caudal and thoracic vertebral and splanchnocranial malformations in a Holstein heifer

A six-month-old Holstein heifer (case 15) was referred to the Institute of Genetics, Vetsuisse Faculty, University of Bern due to congenital microphthalmia and spinal malformations. The heifer was the result of an artificial insemination of a purebred Holstein sire on a Holstein dam. The parents were not related within at least four generations. An on-farm clinical examination was performed. A blood sample was tested for BVDV using ELISA and PCR for detecting antibodies and antigens respectively and both tests resulted to be negative.

The heifer showed a scoliosis of the tail (so-called “crooked tail”) (Supplementary Figure S6, A and B). The spinal processes of the last thoracic vertebra were slightly flat and concave. In

this region, the vertebra seemed to be curved and deviating from the median line of the spine. The viscerocranium was symmetrically narrow (Supplementary Figure S6, B). The heifer revealed a reduced size for her age, but the nutritional status was good. The right eye had a cloudy area in the lens with a diameter of about 4 mm. At neurological examination, the cutaneous pannicularis reflex was absent corresponding to in the region where the thoracic vertebrae were malformed. Other spinal reflexes and the cranial reflexes appeared normal. Considering the clinical findings the clinical diagnosis was compatible with caudal and thoracic vertebral and viscerocranial malformations.

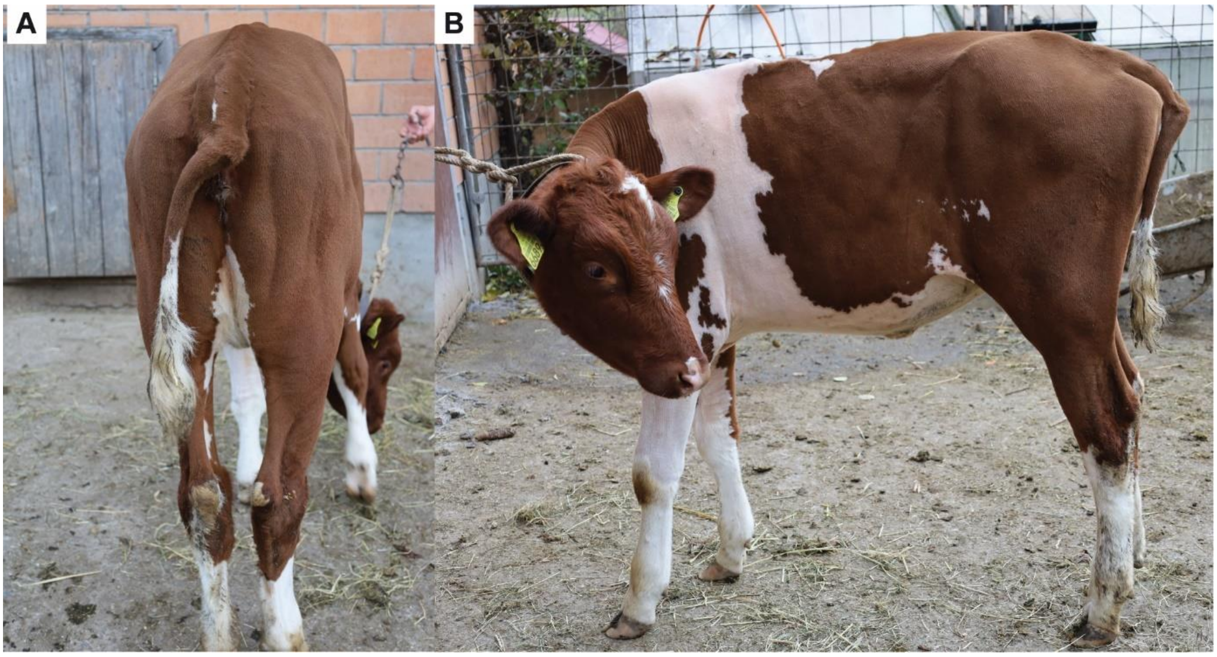

Supplementary Figure S6. Caudal and thoracic vertebral and viscerocranial malformations in a Holstein heifer. **(A)** and **(B)** Note the crooked tail deviated to the left side and a narrow viscerocranium. Case 15.

Primordial dwarfism and slender bones group

Primordial dwarfism in cattle

Primordial proportionate dwarfism in Simmental cattle

A 20-day-old male Simmental calf (case 16) was referred to the Clinic for Ruminants of the University of Ljubljana, Slovenia due to short stature. The calf was born at gestation day 285 as the results of an artificial insemination of an 85% pure Simmental sire on a Simmental dam. The parents were not related within at least four generations. The cow had previously given birth to healthy calves sired by two different sires. One-month post-calving, the cow tested

seronegative for antibodies against BVDV and SBV. An on-farm clinical examination was performed. At the clinical examination, the calf presented a notable short proportionate stature and a slight left tarsus valgus (Supplementary Figure S7, A and B). The nutritional status was good. The calf was bright, alert, and responsive. No other abnormalities were detected.
A second clinical examination was performed on farm at age of three months. The calf continued to present a short proportionate stature.

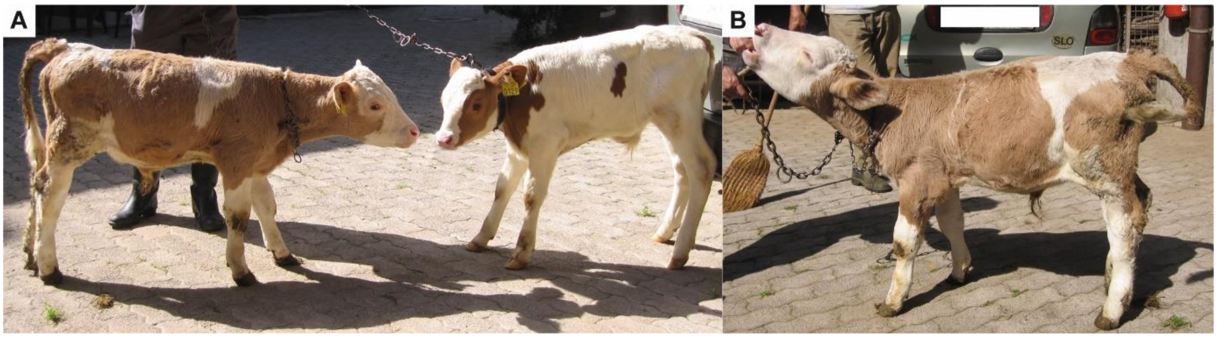

Supplementary Figure S7. Primordial proportionate dwarfism in a Simmental calf. **(A)** Comparison between a 3-month-old proportionate dwarf Simmental calf (left) to a normal 2-week-old Simmental calf (right). Note the almost equal size. **(B)** Proportionate dwarf at age of 3 months. Case 16.

A 20-months-old Simmental heifer (case 17) was referred to the Clinic for Ruminants of the University of Bologna due to short stature. The owner said that the animal showed a small body size since birth. The parents were not related within at least four generations. An on-farm clinical examination was performed. An EDTA-blood sample was tested for BVDV using PCR and ELISA for detecting antigens and antibodies and both tests resulted to be negative. At the clinical examination, the heifer presented a BW of 250 kg, a height at withers of 1 meter and all parts of the body were proportionately small for the age. No other abnormalities were noticed. The animal was clinically diagnosed with primordial proportionate dwarfism.

Cases 16 and 17 were not related within at least 4 generations.

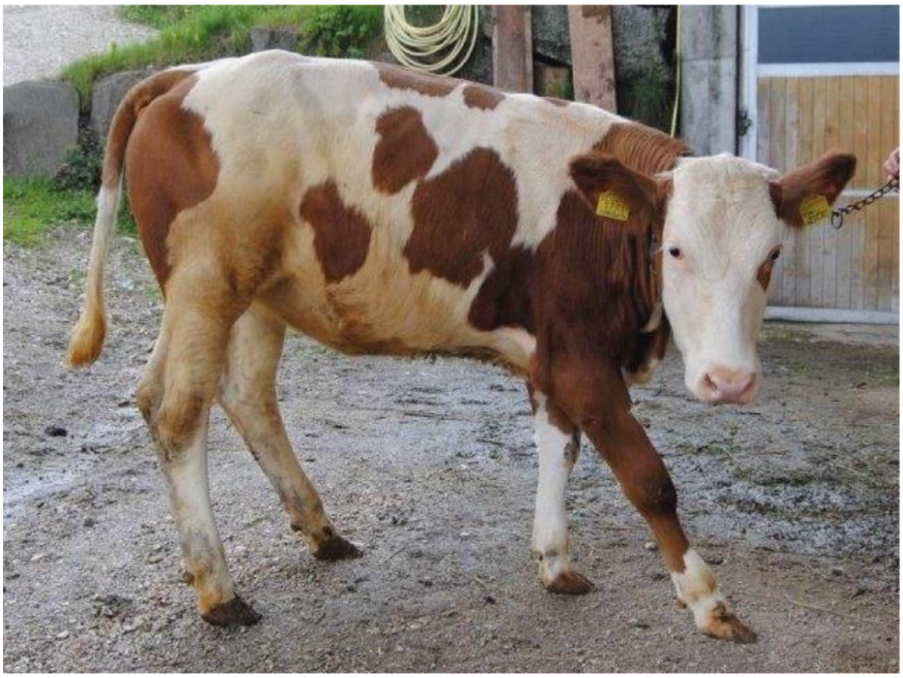

Supplementary Figure S8. Primordial proportionate dwarfism in a Simmental heifer. Case 17.

Primordial disproportionate dwarfism in Angus cattle

A 10-days-old Angus male calf (Case 18) and a 7-months-old heifer (Case 19) were referred to the Farm Post Mortems LDA and SRUC Veterinary and Analytical Services, UK due to short stature. These animals were progeny of the same Angus sire. In case 18, manganese liver levels were measured and were within the normal range (Mn=276.6 µmol/kg DM). Case 19 had a history of metacarpal fracture. Morphologically, the cases showed generalized disproportionate dwarfism and joint laxity. Histology of the ribs was characterized by disorderly hypertrophic chondrocytes columns and similarly irregular narrow hypertrophic zone. Throughout the width of the metaphysis, the primary spongiosa and secondary spongiosa were markedly disorderly with abundant cross linking amphophilic matrix sometimes forming horizontal thick rafts, and columns and disorderly clusters of degenerate chondrocytes extend throughout the metaphysis. The distal femur and tibia displayed shortening of proliferative and particularly of hypertrophic cartilage zones with a remarkably narrow very irregular primary spongiosa with abundant amphophilic matrix. These findings were consistent with an ongoing defect in physeal chondrocyte differentiation and modelling of physeal cartilage matrix and thus supported an intrinsic congenital defect. Taking these findings into account the cases were histologically diagnosed with a form of chondrodysplasia leading to primordial disproportionate dwarfism.

Primordial disproportionate dwarfism in Holstein cattle

A 12-days-old male Holstein calf (case 20) and a 1-day-old male Holstein calf (case 21) were referred to the Department of Animal Breeding and Genetics, Justus Liebig University Giessen, Germany due to congenital short stature. The calves were born in the same year in a commercial dairy farm. The cases were not related within at least three generations. The calves were the result of insemination with semen of a purebred Holstein sires on a Holstein dams. An on-farm clinical examination was performed. EDTA-blood samples were tested for BVDV, SBV and BTV using PCR. Tests were negative. At the clinical examination, case 20 presented a disproportionate short stature with limb shortening (Supplementary Figure S9, A). Additionally, the hindlimbs showed hyperextension of the joints, leading to dropped fetlock. The clinical features were compatible with a form of disproportionate dwarfism. Case 21 showed also disproportionate short stature with limb shortening (Supplementary Figure S9, B). The hindlimbs are endorotated, whereas the forelimbs show a slight exorotation. Furthermore, the calf displayed brachygnathia superior and an enlarged and dome-shaped skull. Case 21 was clinically diagnosed with a primordial syndromic disproportionate dwarfism.

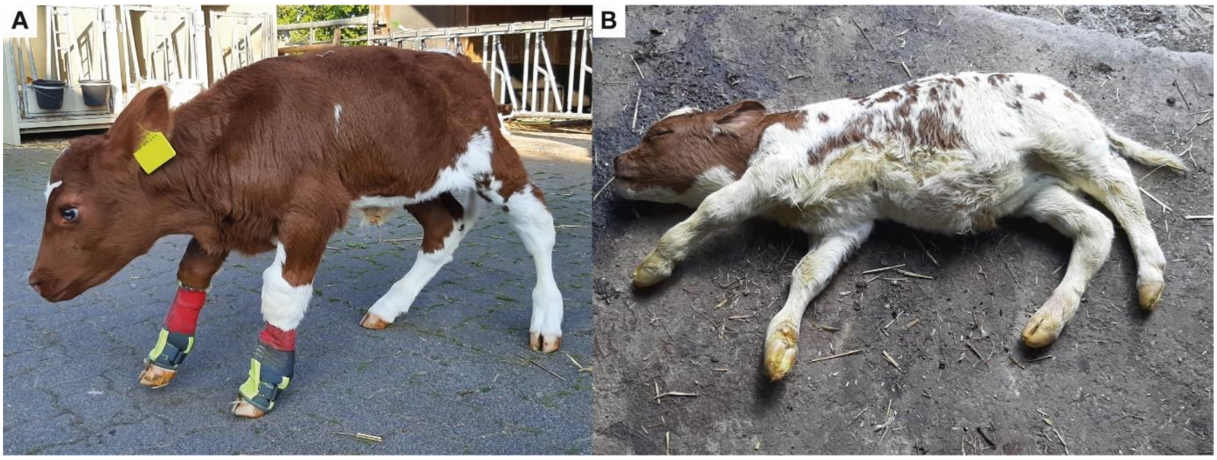

Supplementary Figure S9. Primordial disproportionate dwarfism in Holstein cattle. Note disproportionate short stature with limb shortening case 20 in **(A)** and case 21 in **(B)**.

Primordial proportionate dwarfism in a crossbred calf

A 4-months-old, crossbred (Limousin x Brown Swiss) female calf (case 22) was referred to the Institute of Genetics, Vetsuisse Faculty, University of Bern due short stature. The calf was the result of a natural mating between a Brown Swiss dam and a Limousin sire. The owner referred that the calf was born at term and weighted approximately 30 kg and since then it always presented a good health status but was smaller than the other calves from the same age. An on-farm clinical examination was performed where the heifer presented a BW of 80 kg, slight

brachygnathia and short stature (Supplementary Figure S10). No other abnormalities were noticed. The animal was clinically diagnosed with primordial proportionate dwarfism.

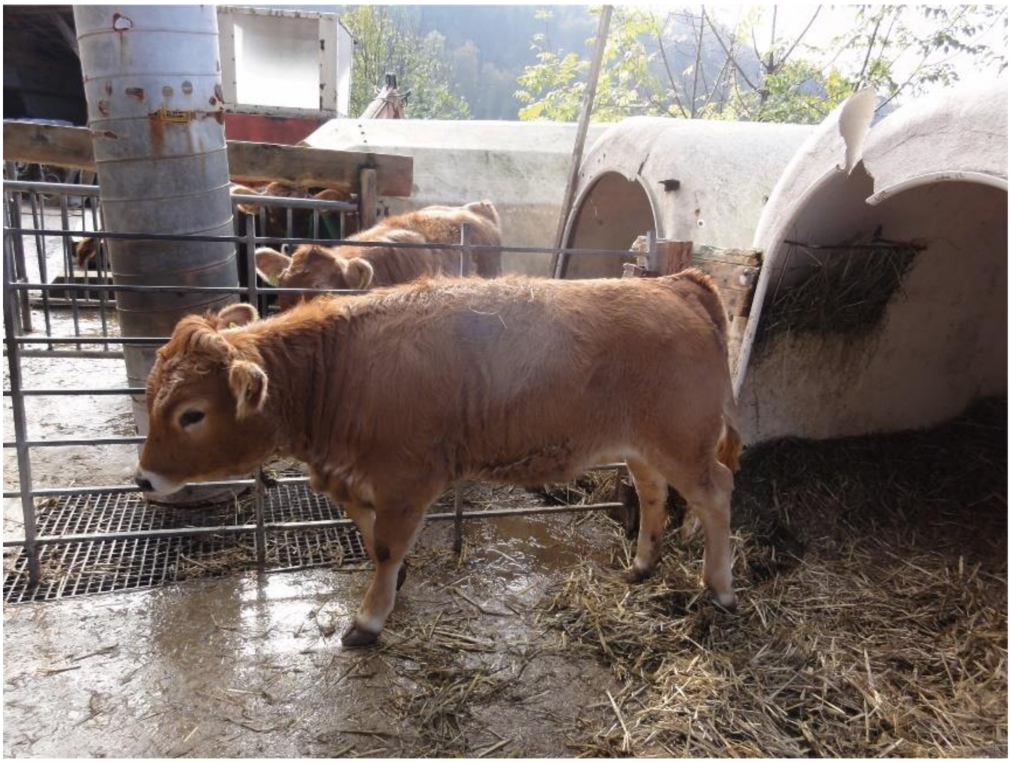

Supplementary Figure S10. Primordial proportionate dwarfism in a crossbred calf. Note the difference between the proportional dwarf calf at four months of age (first plan) compared to healthy one-month-old calves (second plan).

Arachnomelia in a Holstein calf

A stillborn female Holstein calf (case 23) was submitted to the Clinic for Ruminants of the University of Bologna for post-mortem examination. The calf was the result of artificial insemination of a purebred Holstein sire on a Holstein dam. The parents were not related at least within three generations. The calf had abnormally long limbs (dolichostenomelia), angular deformities more pronounced in the forelimbs distal to the carpal region characterized by marked bilateral hyperextension of the carpus bilaterally (Supplementary Figure S11, A and B). Moreover, all fetlocks showed marked bilateral hyperextension and were stiff (Supplementary Figure S11, B). At the radiologic examination of the hindlimbs, the long bone revealed decreased diaphyseal thickness specially the metatarsus. Muscular atrophy of the limbs and flattening of the viscerocranium were noticed. The condition resembled a form of arachnomelia.

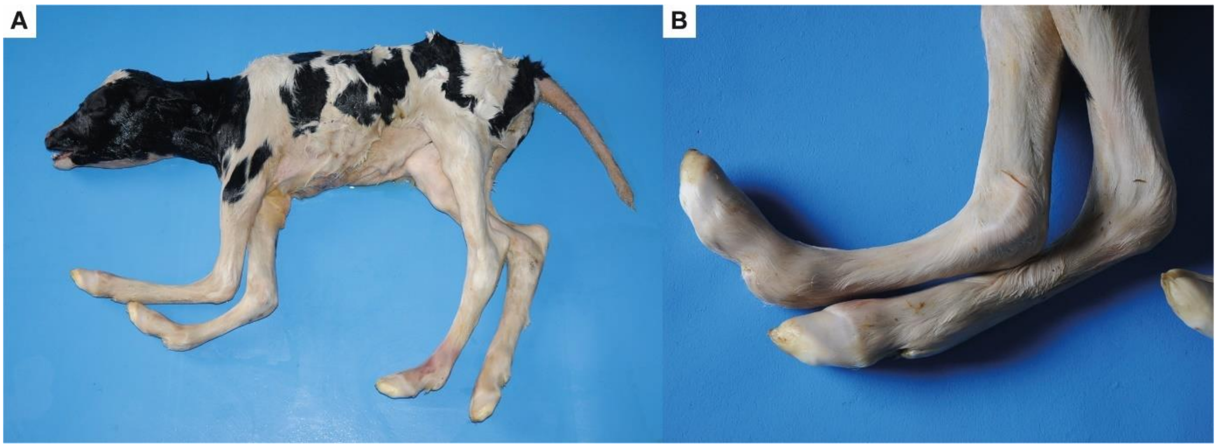

Supplementary Figure S11. Arachnomelia in a Holstein calf. **(A)** Note the dolichostenomelia and angular deformities of all limbs. **(B)** Note the more pronounced angular deformities in the distal forelimbs and marked bilateral hyperextension of the fetlocks. Case 23.

Craniofacial dysmorphism-hydrocephalus-dwarfism syndrome in an Angus calf

A male Angus calf (case 24) with a BW of 25 kg was aborted at gestation day 250. The pregnancy was the result of the insemination of a purebred Angus sire on a purebred Angus dam. The calf was submitted to the Institute of Genetics, Vetsuisse Faculty, University of Bern due to dwarfism. At radiologic examination, the neurocranium was dome shaped. The viscerocranium was markedly shortened. The mandible extended ventrally over the soft tissues of the upper jaw. The premolars of the upper jaw were crowded within its very caudal part. At gross pathology the calf showed superior brachygnathism, clefted palate and deviation of the viscerocranium (Supplementary Figure S12, A and B). Bilateral exophthalmus was observed. The limbs showed bilateral symmetric shortening and widening and were slightly curved (Supplementary Figure S12, A). The lateral ventricles were moderately dilated with serosanguinous fluid compatible with hydrocephalus. At histopathology of the bones, the hypertrophic zone of the growth plate appeared diffusely slightly shortened, but the chondrocytes were physiologically arranged and regular. The findings were consisted with a craniofacial dysmorphism -hydrocephalus-dwarfism syndrome.

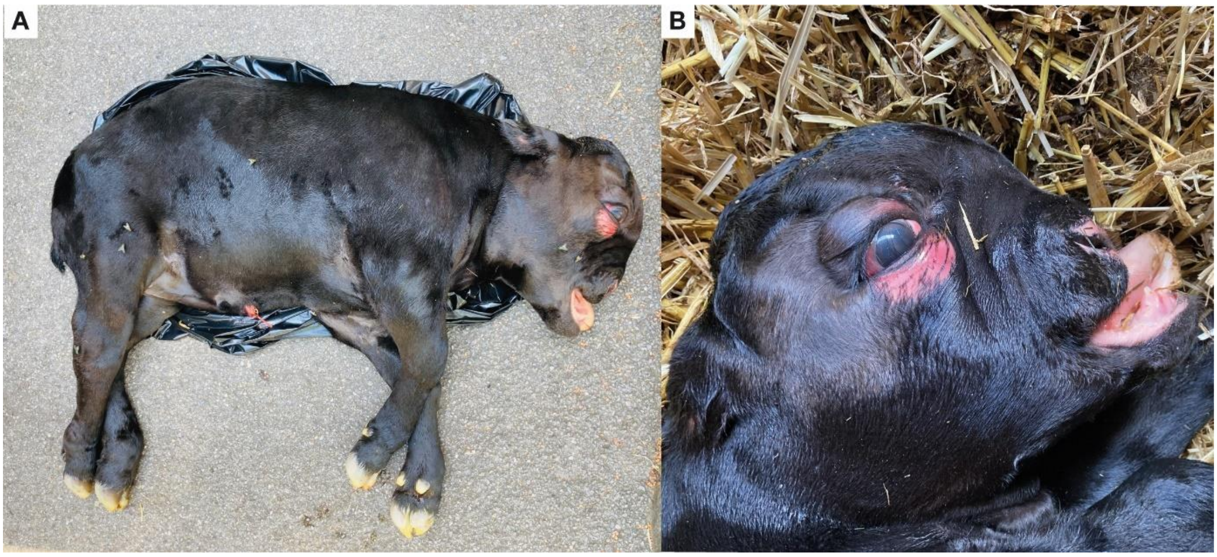

Supplementary Figure S12. Craniofacial dysmorphism-hydrocephalus-dwarfism syndrome in an Angus calf. **(A)** Note the superior brachygnathism, the dome shaped neurocranium, exophthalmos and shortening and widening of the limbs. **(B)** Particular of the head. Note superior brachygnathism, the dome shaped neurocranium, and exophthalmos. Case 24.

Polydactyly-Syndactyly-Triphalangism group

Polydactylism in a crossbred calf

A female crossbred (Inra 95 x Holstein) full-term stillborn calf (case 25) with a BW of 31 kg was aborted. The pregnancy was the result of the insemination of a Inra 95 sire on a purebred Holstein dam. The calf was submitted to the Veterinary Practice Zettlitz, Germany and subsequently to the Saxon State Laboratory for Health and Veterinary Affairs, Leipzig, Germany due to abortion and polydactylism of the forelimbs (Supplementary Figure S13, A). The abaxial wall of the medial digit of the left front foot curled distal and plantar under the foot with a small, extra digit next to the medial digit. The right front foot digits grew divergent with a wide interdigital space. The left forelimb appeared with a duplication at the carpal level. At radiologic examination of the left forelimb, two metacarpi were present (Supplementary Figure S13, B). Distally to the metacarpi, syndactylism was observed bilaterally. Additionally, a ventricular septum defect with a diameter of 0.5 cm was found. The findings were consisted with polydactylism of the forelimbs.

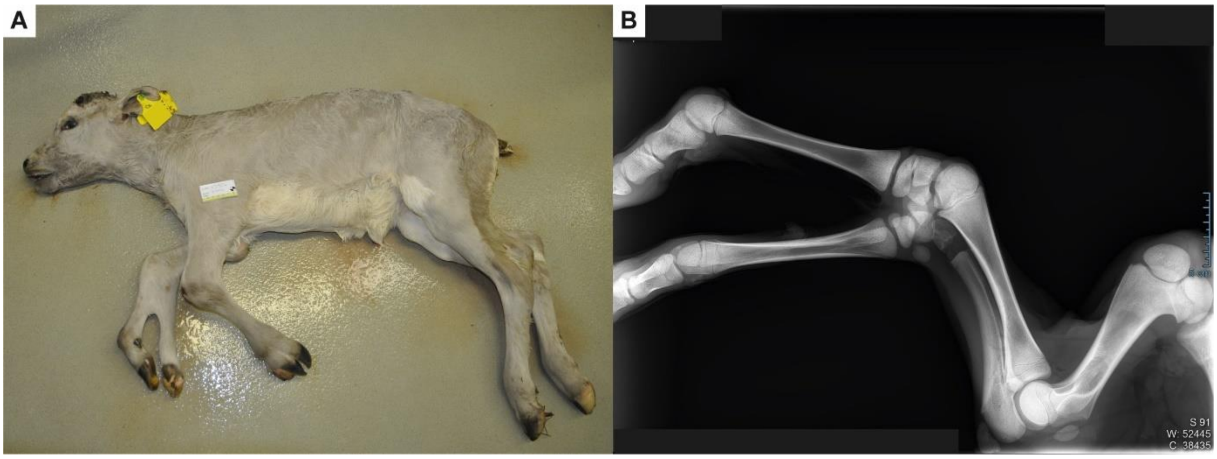

Supplementary Figure S13. Polydactylism in a crossbred calf. **(A)** Note the abaxial wall of the medial digit of the left front foot curled distal and plantar under the foot with a small, extra digit next to the medial digit and the left forelimb shows a duplication at the carpal level. **(B)** Radiograph of the right forelimb showing a duplication of the limb at the carpal level. Note the presence of two metacarpi.

Syndactylism in Holstein cattle

Three Holstein calves (Cases 26 – 28; two females and one male) presented syndactyly of one forelimb characterized by the fusion of the two distal phalanges (synostotic phalanges). Cases 26 and 28 were second generation relatives on the paternal side, while case 28 was not related to cases 26 and 27 within at least three generations.

Syndactyly in a Droughtmaster calf

A six-month-old Droughtmaster male calf (case 29) was referred to the Elizabeth Macarthur Agricultural Institute, NSW Department of Primary Industries and Regional Development, Australia because of syndactylism. The animal showed syndactylism on both front feet characterized by the fusion of the two distal phalanges (synostotic phalanges) and partial fusion of the two distal phalanges in the left forelimb (Supplementary Figure S14). Both back feet were normal.

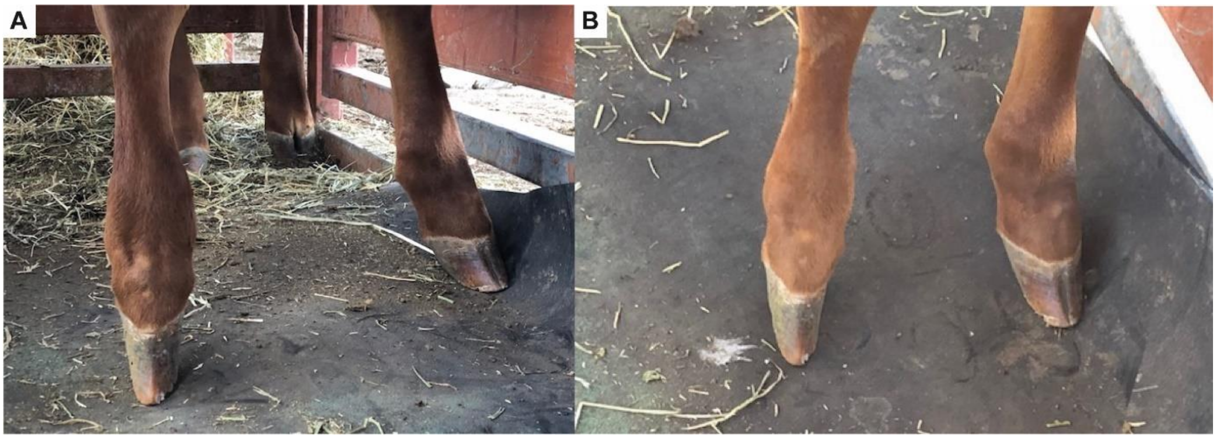

Supplementary Figure S14. Syndactyly in a Droughtmaster calf. **(A)** and **(B)** Note the syndactyly of the right forelimb and the partial syndactyly in the left forelimb. Boths back feet are normal. Case 29.

Disorders of bone mineralization

Congenital rickets in Angus cattle

Two full-term paternal half-siblings female Angus calves (cases 30 and 31) died shortly after birth and were submitted to the APHA. The calves showed similar gross pathology findings which included shortening of the bones of the hindlimbs and cerebellar herniation both calves. Histologically, the long bones were characterized by plentiful cartilage nodules, often with pale eosinophilic (degenerate) matrix, extending into the subarticular bone and metaphysis, the latter associated with disorderly primary spongiosa and irregular physeal cartilage particularly hypertrophic zone. Bone spicules with minimal remodeling, thus mainly perpendicular to the growth plate, continued deep into the diaphysis. The ribs showed very disorderly expanded physis in both calves with very irregular elongated hypertrophic cartilage zone and retention of irregular cartilage cores in irregular trabeculae in the metaphysis and deep into the diaphysis, some with pale eosinophilic (degenerated) matrix in one calf which had abundant fibrin and erythrocyte extravasation at the physeal / metaphyseal junction. These alterations were considered consistent with congenital rickets, especially due to the changes in the subarticular bone, and more marked involvement of the rib.

Dysplasias with multiple joint dislocations

Craniofacial dysmorphisms and forelimb skeletal dysplasia in Holstein

A stillborn male Holstein calf (case 32) was submitted to the University of Copenhagen for postmortem examination as part of the Danish surveillance program for bovine genetic diseases

due to the presence of forelimb malformation. The calf was born at gestation day as the result of an artificial insemination of a purebred Holstein sire on a Holstein dam. The parents were not related within at least four generations.

The calf was of normal size with a body weight of 32.9 kg. The forelimbs were dysplastic and had bilateral almost symmetric arthrogryposis with an almost 90° medial rotation if the carpal joint (Supplementary Figure S15, A). The two main digits and the medial dewclaw were developed in the left forelimb, while only the medial digit and the corresponding dewclaw were present in the right forelimb.

Both thoracic limbs were selected for radiology. The most striking lesion was absence of the radius, but additional multiple abnormalities in bone development were observed (Supplementary Figure S15, B). The shoulder joint was luxated and the ulnar notch poorly developed with minimal contact between the ulna and humeral condyle. The ulnar mid diaphysis was thickened, and its distal part was expanded to form a broad-based articulation with the carpus. The radiocarpal joint was misaligned. Metacarpal III was present and articulated distally with normal appearing phalanges. A short thin bone structure (0.3 cm diameter by 1.75 cm length) was present adjacent to proximal metacarpal III and was interpreted as a rudimentary metacarpal IV.

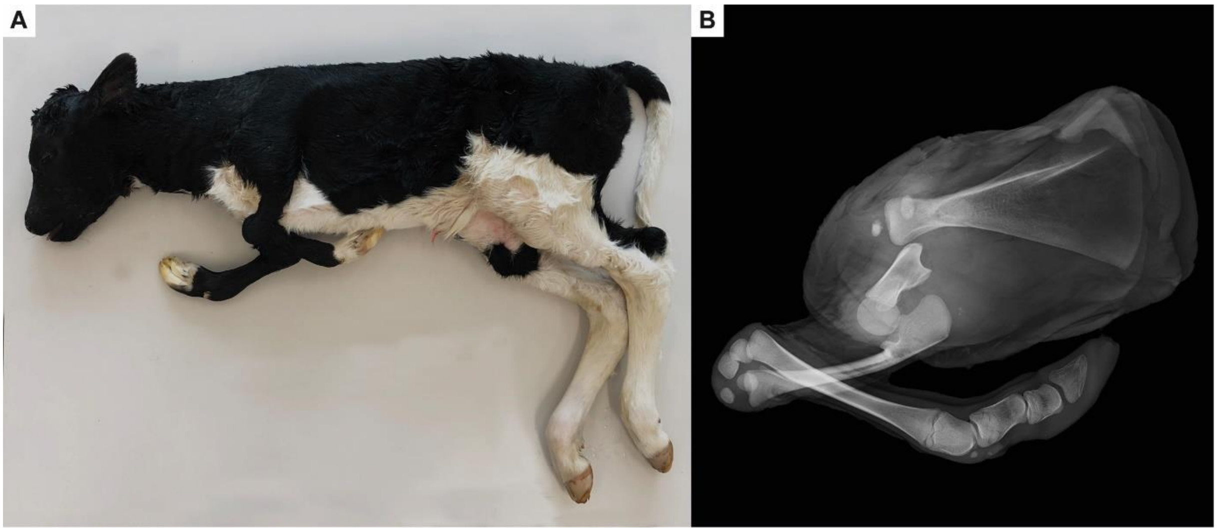

Supplementary Figure S15. Craniofacial dysmorphisms and forelimb skeletal dysplasia in a Holstein calf. **(A)** Note that both forelimbs are dysplastic with bilateral almost symmetric arthrogryposis and medial rotation if the carpal joint. The two main digits and the medial dewclaw were developed in the left forelimb, while only the medial digit and the corresponding dewclaw were present in the right forelimb. **(B)** Radiograph of the right forelimb showing absence of the radius. Additionally, luxation of the shoulder joint a poorly developed ulnar

notch, there is minimal contact between the ulna and humeral condyle, ulna's mid diaphysis is thickened and in its distal part it is expanded to form a broad based articulation with the carpus, metacarpal III is present and articulates distally with normal appearing phalanges and a short thin bone structure (0.3 cm diameter by 1.75 cm length) is present adjacent to proximal metacarpal III; this is presumed to be a rudimentary metacarpal IV. Case 32.

Craniofacial dysmorphisms, forelimbs dislocations and skeletal dysplasia in a Holstein calf

A 15-days-old male Holstein calf (Case 33) was referred to the Clinic for Ruminants and Herd Health Management of the Justus Liebig University Giessen due to congenital malformation of the limbs. The calf was recovered in the clinic, and complete clinical and pathological examinations were performed. The calf showed axial limb malalignment (Supplementary Figure S16, A and B). The glenohumeral joints were rotated by approximately 45° laterally. The forelimbs were rotated medially, with the limbs crossed over in the region of the body, resting on the carpus. The hindlimbs were rotated laterally and showed valgus position, especially in the tarsus. The joints were moderately stiff. Additionally, there was a slight brachygnathia superior and brachycephaly. The breathing was costoabdominal at with a respiratory rate of 100 rpm. Thoracic auscultation revealed slightly increased inspiratory sounds. Heart rate was 136 bpm and temperature was 38.5°C. The feces were brown-grey and malodorous.

Blood hematology and biochemistry revealed reduced cholesterol [1.7 mmol/l, reference range (RR:) 2.07–3.88 mmol/l] and total protein (53.5 g/l, RR: 60–80 g/l). Triglycerides (0.93 mmol/l, RR: 0.17–0.51 mmol/l), inorganic phosphorus (2.6 mmol/l, RR: 1.1–2.4 mmol/l) and potassium (5.5 mmol/l, RR: 3.5–4.5 mmol/l) were slightly to moderately elevated. Glucose (7 mmol/l, RR: 1.94–3.05 mmol/l) and iron (98.7 µmol/l, RR: 20–40 µmol/l) levels were significantly elevated. Microscopic flow cytometry and differential blood count (absolute numbers) showed a slightly elevated hematocrit (0.39 l/l, RR: 0.28–0.38 l/l) and highly elevated leukocyte (27.9 G/l, RR: 4.0–10.0 G/l) and platelet counts (951 G/l, RR: 300–800 G/l). A test for hypochromasia was positive. Segmental nuclei were elevated by 73 % (20.4 G/l, RR: 1.0–3.5 G/l) and eosinophils by 14 % (3.9 G/l, RR: 0.3–1.5 G/l). In addition, vitamin D level was reduced to 36.73 nmol/l (RR: 75–125 nmol/l). The reference ranges and measurements are given according to the International Federation of Clinical Chemistry specifications.

A radiologic examination of the limbs was performed. The left forelimb showed moderate axial rotation from the distal radius diaphysis laterally (exorotation). The right anterior limb showed an oblique scapular fracture with subtle dislocation medio-proximally in the reparation phase,

but with significant callus formation. In addition, there was a high degree axis rotation, again from the distal radius diaphysis laterally (exorotation). Both hindlimbs had a high degree axial deviation at the level of the proximal tibial apophysis to lateral, with a convex curvature to medial. The significant axis rotation occurred medially starting at this point (endorotaion) (Supplementary Figure S16, C). Tibia and fibula showed a distinct medial convex curvature of approximately 46.5 °. The condition was compatible with craniofacial dysmorphisms, forelimbs dislocations and skeletal dysplasia.

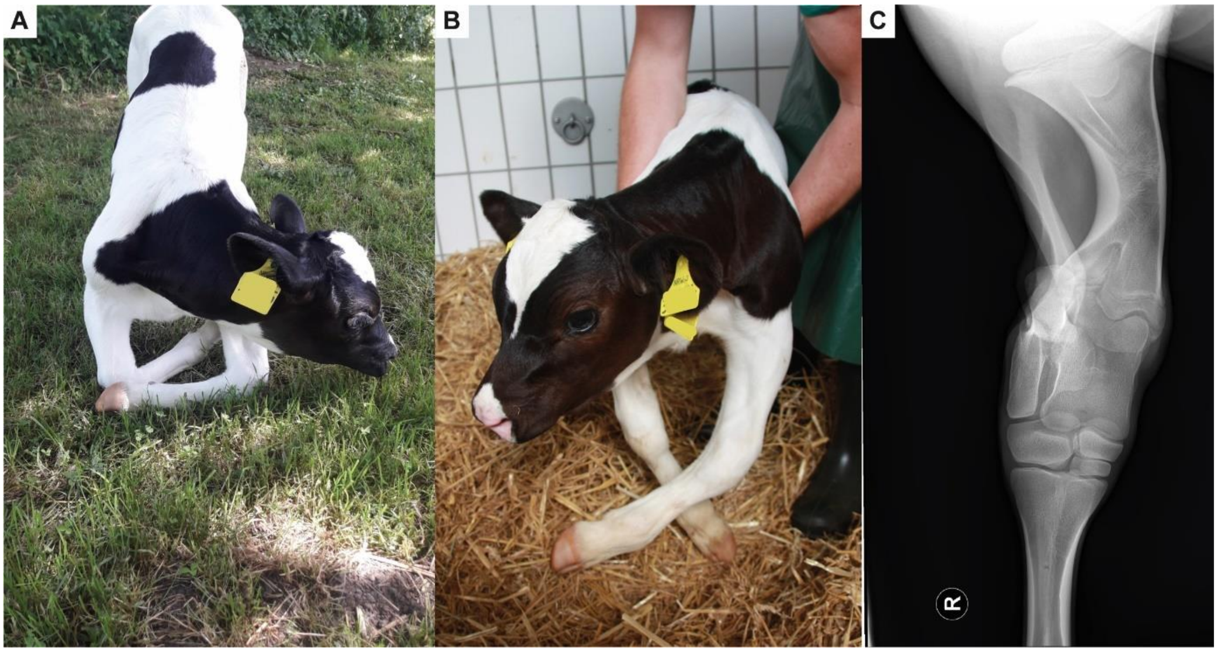

Supplementary Figure S16. Craniofacial dysmorphisms, forelimbs dislocations and skeletal dysplasia in a Holstein calf. **(A)** and **(B)** Note axial limb malalignment and the lateral rotation of the radial bones of both forelimbs. **(C)** Dorsoplantar radiography of the right hindlimb. Note the prominent endorotation of the tibia starting proximal at the tibial apophysis with medial curving of the tibial diaphysis. Axial rotation results in a malalignment of the femoro-tibial and tarso-metatarsal joints. Case 33.

Craniofacial dysmorphism and forelimbs dysplasia with joint contracture in a Chianina heifer

A 19-months-old female Chianina heifer (Case 34) was referred to the Clinic for Ruminants of the University of Bologna due to poor general condition and skeletal malformations. The heifer was the result of an artificial insemination of a purebred Chianina sire on a Chianina dam.

A clinical examination on farm was perform. The heifer presented an anomalous generalized skeletal development craniofacial dysmorphism including shortening of the viscerocranium, concavity of the nasal bone, brachygnathia superior and protrusion of the tongue

(Supplementary Figure S17, A and B). The tongue had a physiological shape and tone even though it was continuously moved with ‘snake-like’ movements. The forelimbs showed bilateral flexural deformities characterized by lateral rotation and bowing with joint swelling and loss of movement (join contracture) (Supplementary Figure S17, A). The clinical findings were compatible with craniofacial dysmorphism and forelimbs dysplasia with joint contracture.

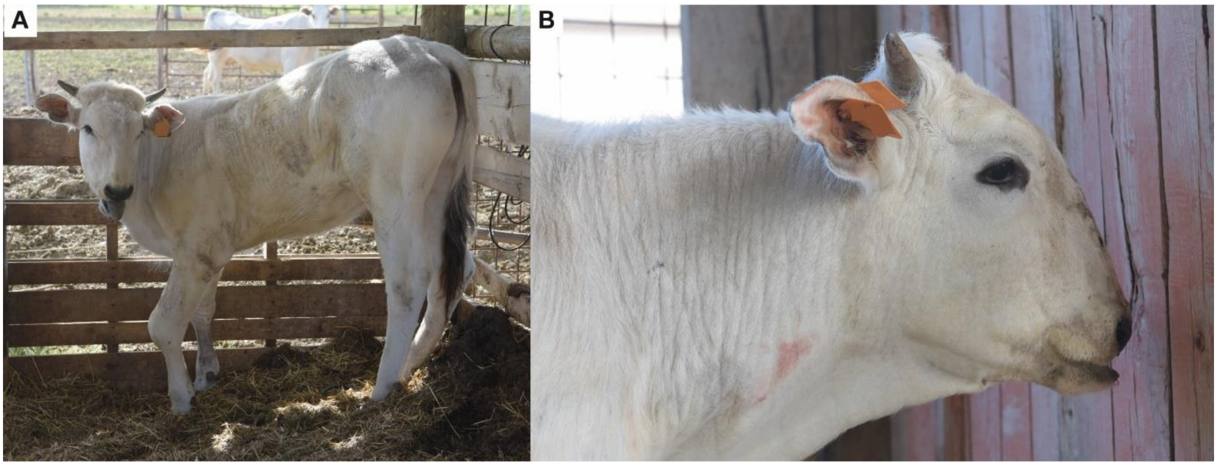

Supplementary Figure S17. Craniofacial dysmorphism and forelimbs dysplasia with joint contracture in a Chianina heifer. **(A)** Note the craniofacial dysmorphism, protrusion of the tongue and the bilateral flexural deformities of the forelimbs characterized by lateral rotation and bowing with join contracture. **(B)** Particular of the head. Note the craniofacial dysmorphism including shortening of the viscerocranium, concavity of the nasal bone, brachygnathia superior. Case 34.

Forelimbs dysplasia with joint contracture in a Holstein calf

A 5-days-old female Holstein calf (Case 35) was referred to the Department of Animal Breeding and Genetics, Justus Liebig University Giessen due to congenital malformation of the forelimbs. A clinical examination was performed on farm. EDTA-blood samples were tested for BVDV, SBV and BTV using PCR. Tests were negative. The calf was not able to stand after birth. According to the breeder, it was due to the exorotation of the shoulders and simultaneous endorotation of the forelimb. The forelimb joints were stiff. The physiologic condition improved some weeks after birth. The calf was not necropsied.

Forelimbs dysplasia with joint contracture in a Chianina heifer

A 16-months-old female Chianina heifer (Case 36) was referred to the Clinic for Ruminants of the University of Bologna due to poor general condition and skeletal malformations. The heifer was the result of an artificial insemination of a purebred Chianina sire on a Chianina dam. A on-farm clinical examination was performed. The heifer showed retarded growth, bilateral

elbow abduction and flexor deformity on the carpus bilaterally (carpus valgus) (Supplementary Figure S18, A and B). The shoulder muscles were hypotrophic but normotonic. The carpus joint was partially ankylosed and flexed. On palpation there were no painful, swollen or warm joints. Serological and direct tests for BVDV, SBV and BTV were negative. Blood biochemistry revealed elevated serum phosphorus concentrations (8.2 mg/dl, RR: 5,6–6.5 mg/dl).

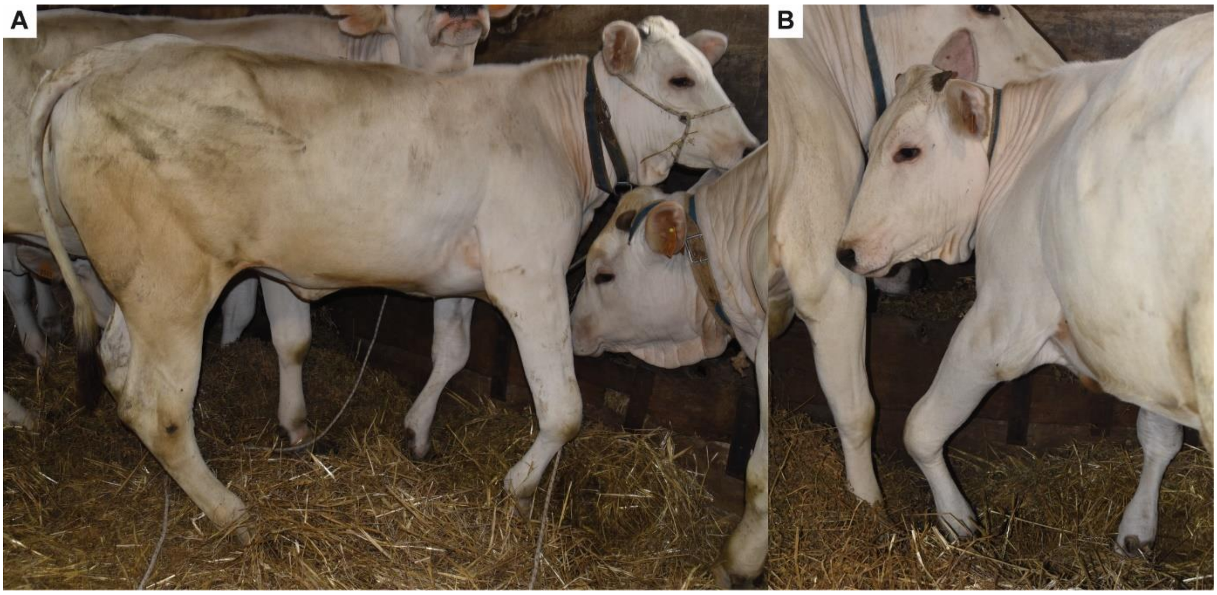

Supplementary Figure S18. Forelimbs dysplasia with joint contracture in a Chianina heifer. **(A)** and **(B)** Note the bilateral elbow abduction and flexor deformity on the carpus bilaterally.

Forelimbs dysplasia with joint contracture in Limousin calves

Two paternal half-siblings male Limousin calves (cases 37 and 38) from the same farm were referred to the Institute of Genetics, Vetsuisse Faculty, University of Bern because of unilateral congenital malformation of a forelimb. The owner reported that two other affected calves had been born previously. These were paternal half-siblings of cases 37 and 38. The affected animals were the result of a natural mating between Limousin dams and a Limousin sire. Cases 37 and 38 underwent on-farm clinical examination. Blood samples were tested for SBV using PCR and ELISA for detecting antigens and antibodies, respectively. Tests were negative.

Cases 37 and 38 presented an abnormal posture of the left forelimb. The left carpus was flexed at an angle of approximately 150° and 130° with medial deviation (carpus valgus) in cases 37 and 38, respectively (Supplementary Figure S19). The calves were mildly lame with a shortened stride. At palpation the left carpal joint of both affected animals was non-warm, non-painful, with normal consistency. To the passive mobility it was possible to flex and extend the carpus without resistance. Case 37 showed asymmetry of the muzzle and a slight inferior prognathism. No other abnormalities were retrieved at the clinical examination.

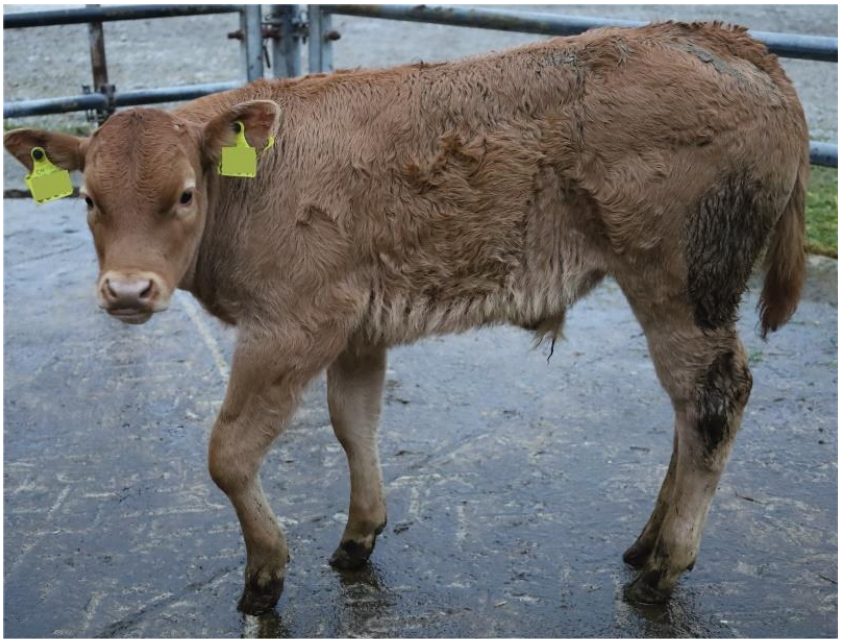

Supplementary Figure S19. Forelimbs dysplasia with joint contracture in Limousin calves. Note the carpus valgus. Case 38.

Vertebral and costal dysostoses

Hemifacial microsomia with hemivertebrae in a Rendena calf

A 7-day-old male Rendena calf (case 39) with a body weight (BW) of 30 kg was referred to the Ruminant Clinic of the University of Bologna for congenital facial asymmetry and weakness. The owner reported that this was the fourth calf with these characteristics in the last five years. The calf was the result of artificial insemination of a purebred Rendena sire on a Rendena dam. The parents were not related for at least three generations. On clinical examination, the calf had a particularly short neck, scoliosis, brachygnathia, unilateral microtia (left external auricle) and facial asymmetry with deviation of the median plane to the left (Supplementary Figure S20, A and B). The animal's ears were pendulous, pointing downward more on the left side. There was also apparent atrophy of the masseter muscles and flaccidity of the left lip (drooping of the mouth). The animal had a divergent strabismus in the left eye. The nutritional status was poor, with reduced appetite and dysphagia. The calf showed reduced mentation and sensory perception, with almost no interaction with the environment, in particular no response to auditory stimuli. In quadrupedal stance, the animal kept its head low and moved its neck horizontally from side to side continuously and repeatedly. The calf exhibited gait abnormalities characterized by slow and short steps, with the dorsum and claws dragging on the ground during the step. In addition, the calf tended to walk in a circle to the left (circling gait). The palpebral

and corneal reflexes were present bilaterally. Response to facial nerve stimulation was present but reduced bilaterally. The auricular reflex appeared to be reduced bilaterally. The calf temperature was 38.9°C, the pulse was not palpable, and the respiratory rate was 86 breaths per minute, predominantly abdominal. Cardiac auscultation revealed tachycardia (120 beats per minute) and arrhythmia, but no murmurs.

Due to the poor prognosis, the calf was euthanized after clinical examination. Gross pathology showed that the left half of the diaphragm was completely aponeurotic and displaced cranially, whereas the right half was physiological. The right testicle (dimension: 2.5x2x1cm) was located dorsocranially to the right kidney, whereas the left testicle (dimension: 3x1.5x1 cm) was located in the scrotal sac. The presence of 14 ribs was noted on the left side, with fusion of the second and third ribs. On the right side there were 13 ribs, of which the first, second and the most dorsal part of the third rib were fused. The vertebrae articulating with the above ribs were also fused. A grade IV ectopia of the spiral loop of the ascending colon was noted. There were multiple small (up to 0.5 cm in diameter) abscesses in the cranial lobes and multifocal atelectatic areas in the caudal lobes of both lungs. There was also hypoplasia of the right caudal lobe. Histological exam of parenchymatous organs demonstrated no injuries.

Brain samples were tested for bovine viral diarrhea virus (BVDV), Schmallenberg virus (SBV) and bluetongue virus (BTV) using PCR. Tests were negative for all these pathogens.

Based on the clinical and pathological findings, the calf was diagnosed with a form of microsomia with hemivertebrae.

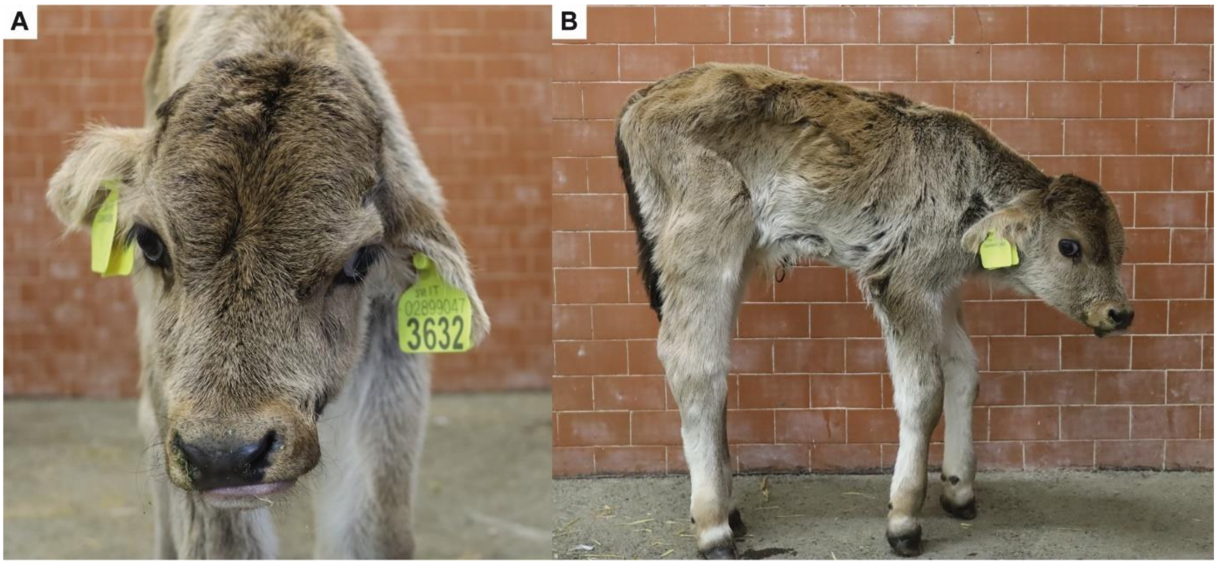

Supplementary Figure S20. Microsomia with hemivertebrae in a Rendena calf. **(A)** Note the unilateral microtia (left external auricle), divergent strabismus in the left eye and facial asymmetry with deviation of the median plane to the left. Note that the ears are pendulous,

pointing downwards more markedly on the left. Note the atrophy of the masseter and flaccidity of the left lip (drooping of the mouth). **(B)** Note the short neck and reduced nutritional status.
